# Changes in brain rhythms and connectivity tracking fear acquisition and reversal

**DOI:** 10.1007/s00429-023-02646-7

**Published:** 2023-05-02

**Authors:** Gabriele Pirazzini, Francesca Starita, Giulia Ricci, Sara Garofalo, Giuseppe di Pellegrino, Elisa Magosso, Mauro Ursino

**Affiliations:** 1 Department of Electrical, Electronic, and Information Engineering “Guglielmo Marconi”, University of Bologna, Area di Campus Cesena, Via Dell’Università 50, 47521 Cesena, Italy; 2 Center for Studies and Research in Cognitive Neuroscience, Department of Psychology “Renzo Canestrari”, University of Bologna, 40126 Bologna, Italy

**Keywords:** Fear conditioning, Reversal, Granger connectivity, Theta rhythm, Alpha rhythm

## Abstract

Fear conditioning is used to investigate the neural bases of threat and anxiety, and to understand their flexible modifications when the environment changes. This study aims to examine the temporal evolution of brain rhythms using electroencephalographic signals recorded in healthy volunteers during a protocol of Pavlovian fear conditioning and reversal. Power changes and Granger connectivity in theta, alpha, and gamma bands are investigated from neuroelectrical activity reconstructed on the cortex. Results show a significant increase in theta power in the left (contralateral to electrical shock) portion of the midcingulate cortex during fear acquisition, and a significant decrease in alpha power in a broad network over the left posterior-frontal and parietal cortex. These changes occur since the initial trials for theta power, but require more trials (3/4) to develop for alpha, and are also present during reversal, despite being less pronounced. In both bands, relevant changes in connectivity are mainly evident in the last block of reversal, just when power differences attenuate. No significant changes in the gamma band were detected. We conclude that the increased theta rhythm in the cingulate cortex subserves fear acquisition and is transmitted to other cortical regions via increased functional connectivity allowing a fast theta synchronization, whereas the decrease in alpha power can represent a partial activation of motor and somatosensory areas contralateral to the shock side in the presence of a dangerous stimulus. In addition, connectivity changes at the end of reversal may reflect long-term alterations in synapses necessary to reverse the previously acquired contingencies.

**Supplementary Information:**

The online version contains supplementary material available at 10.1007/s00429-023-02646-7.

## Introduction

Fear conditioning is a paradigm used in neuroscience to study the neurobiological bases of threat and anxiety (Duits et al. 2015; Starita et al. 2016, 2019a). In this experimental protocol, a neutral stimulus (conditioned stimulus, CS +) is paired with an aversive stimulus (unconditioned stimulus, US) to trigger neural adjustments at the basis of fear acquisition, finally resulting in the expression of a fear response in the presence of the CS + alone. In some experiments, a second conditioned stimulus (CS-) is unpaired with the US to serve as a control condition.

Results in rodents, primates, and humans provide a precise scenario showing the amygdala, hippocampus, and medial prefrontal cortex (mPFC) involvement in fear acquisition (Bechara et al. 1995, 1999; LaBar et al. 1995, 1998; Knight et al. 2005; Cheng et al. 2006; Fullana et al. 2016; Bertini et al. 2020; Battaglia et al. 2020). Furthermore, brain oscillations in the theta range (4–8 Hz) play a pivotal role in this process (Sperl et al. 2019). In rodents enhanced theta synchrony between the amygdala and mPFC has been observed, which differentiated between threat and safety (Likhtik et al. 2014). In rats, behavioral fear expression, like freezing, coincides with internally generated theta oscillations in prefrontal-amygdala circuits (Karalis et al. 2016). In primates, theta power and coherence in the amygdala and anterior cingulate cortex increase during fear learning, but progressively decline once the association is stabilized (Taub et al. 2018). In humans, neuroimaging studies suggest that the recall of conditioned fear involves the anterior midcingulate cortex (AMC), which exhibits more robust activation to fear-conditioned stimuli (Knight et al. 2004; Phelps et al. 2004; Milad et al. 2007a). Source localization from electroencephalographic (EEG) signals reveals that fear-conditioned stimuli evoke significantly more theta activity in the AMC than not fear-conditioned stimuli (Mueller et al. 2014).

Besides theta oscillations increase, a decrease in alpha power (8–14 Hz) is also observable during fear conditioning, especially in the first block of the stimulus train. These changes have been mainly reported in the parietal and occipital channels (Chien et al. 2017; Yin et al. 2020), and seem to reflect the valence (i.e., unpleasantness) and salience (i.e., relevance) of the stimulus, rather than fear conditioning per se. (Bacigalupo and Luck 2022) observed that alpha-band suppression is greater for the CS + compared to the CS- during fear acquisition, and that this effect is reduced during extinction. (Babiloni et al. 2008) suggested that greater alpha power reduction occurs during anticipatory processes preceding the integration of painful and motor information, compared with painful stimuli which do not require motor tasks. (Panitz et al. 2019) analyzed visuocortical alpha suppression in response to conditioned and extinguished threat. They observed an alpha power suppression at the occipital lobes, which was more pronounced for CS + than CS- and survived extinction. The authors interpreted this result as an attentional prioritization of behaviorally more relevant stimuli.

However, to understand fear mechanisms, it is also essential to clarify how this response can be flexibly adjusted depending on varying environmental conditions (Garofalo et al. 2014, 2017; Magosso et al. 2015). Substantial knowledge of this process is provided by extinction protocols, in which a previously conditioned subject is exposed to CS + in the absence of the aversive input. Extinction is characterized by a progressive decrease in the response to CS, while deficits in extinction mechanisms are associated with pathological states like post-traumatic stress disorder and anxiety (Marin et al. 2017; Trenado et al. 2018; Çalışkan and Stork 2019). Neuroimaging studies in humans underline the involvement of the ventromedial prefrontal cortex (vmPFC) in extinction (Etkin et al. 2011; Milad and Quirk 2012). EEG studies demonstrate that theta oscillations in the dorsal anterior cingulate cortex are reduced during successful extinction recall, probably involving an interplay between the amygdala and frontomedial theta activity (Sperl et al. 2019). A pivotal role in extinction seems related to gamma oscillations (> 30 Hz) localized in the vmPFC, since extinguished vs. non-extinguished stimuli evoked an increased gamma power differential response localized in this area (Mueller et al. 2014).

Reversal learning is an alternative way to study flexibility. This protocol is frequently adopted to analyze the classic reward-based action selection (i.e., decision making) involving the dopaminergic system and basal ganglia plasticity (Schirru et al. 2022); however, only a few studies have explored reversal during fear conditioning. During reversal learning, a subject must be able to modify the previously learned stimulus-outcome association by inhibiting a previous response in favor of a new one. Schiller et al. (2008) pointed out fear reversal is more demanding than fear extinction. In fact, during reversal, not only the old aversive stimulus must be extinguished, but also a new stimulus (the old CS-) acquires an aversive value. Hence, using reversal learning, one can examine how a fear response is weakened while another fear response is simultaneously acquired, allowing a concurrent comparison between the two mechanisms and favoring a better understanding of their neurological bases.

There is a large consensus that, during action-selection tasks, reversal learning involves the ventral PFC, especially the orbitofrontal cortex. Increased activation in this area seems to be predominantly associated with unexpected rewards and punishments, thus signaling the need for flexible behavior and playing a fundamental role in action control (Rolls 2004). However, less is known about the reversal of Pavlovian fear. Using functional neuroimaging in conjunction with a fear-conditioning reversal paradigm, Schiller et al. (2008) emphasized the role of the vmPFC, showing that the activity in this region increases during an unexpected safe condition (i.e., during the new CS- in reversal), thus providing a possible reward signal. A similar role for the vmPFC was previously stressed in an fMRI study by (Kim et al. 2006), suggesting that activity in the orbitofrontal cortex increases not only following a reward but also during a successful avoidance of an aversive outcome. However, an opposite result can be found in (Morris and Dolan 2004). In their study, the right orbitofrontal cortex exhibited increased response during CS + than CS-, both in the acquisition and reversal phases.

Although changes in the power of rhythms are well documented during fear conditioning and extinction, as summarized above, we are not aware of studies that examine the variations in these rhythms during a fear reversal paradigm, in particular, by comparing the temporal evolution of theta, alpha, and gamma power when the aversive valence progressively shifts from one stimulus to another. Several aspects need to be further elucidated: (i) What is the role of different brain rhythms during reversal? (ii) How fast can reversal learning occur? (iii) Is the new fearful condition specular to the previous one (i.e., the one acquired before reversal), or do the two fearful conditions exhibit differences in brain activity and rhythms?

The scenario is even more complex if one considers the role of brain connectivity. (Hudson et al. 2020) studied the brain bases of sustained and acute fear using naturalistic fMRI and showed that fear is associated with profound changes in connectivity. Since conditioning implicates changes in synaptic plasticity not only in the hippocampus and amygdala but above all in the cortex and involves the participation of a system of rhythms in various cortical areas (Buzsaki 2006; Wang 2010), it may be of great value to analyze how functional connectivity changes during the acquisition and the reversal phases of a fear conditioning paradigm. (iv) How does functional connectivity modify during fear acquisition in different brain regions and frequency bands? (v) Is connectivity after reversal the specular form of the connectivity obtained in the previous acquisition phase, or does connectivity maintain some reminding of the last state?

Although multiple studies on fear acquisition and extinction have appeared in recent years, we think these questions are not entirely clarified yet. In particular, in the following, we will examine the temporal evolution of the power of brain rhythms and brain connectivity over the course of experimental trials, to fully describe the development of fear during acquisition and its shift from one conditioned stimulus to another during reversal. To this end, we reanalyzed data from a group of healthy participants that completed a fear acquisition and reversal task (Starita et al. 2023). We previously reported that changes in theta and alpha power discriminate between threat and safety and correlate with skin conductance response. Specifically, we found a significant positive correlation between theta-band power and skin conductance response, regardless of the stimulus, and a significant strong negative correlation between alpha-band power and skin conductance response for the CS + . These results underline the existence of a direct relationship between central and peripheral nervous system activation. This study extends those results by examining the changes in theta, alpha and gamma power over the course of experimental trials and brain connectivity obtained from cortical source reconstruction in 76 cortical areas, and estimating Granger connectivity.

## Methods

### Participants

The experiments took place at the Centre for studies and research in Cognitive Neuroscience (CsrCN) of the University of Bologna. Twenty healthy volunteers were recruited. All participants were right-handed and had normal or corrected to normal vision and reported no medical or psychiatric illness. One participant did not complete the experimental session because of a fainting and was excluded from the dataset. Thus, nineteen participants have completed the study (8 males, mean age = 23.48, std = 1.85). The study followed the American Psychological Association Ethical Principles of Psychologists and Code of Conduct and the Declaration of Helsinki and was approved by the local Bioethics Committee of the University of Bologna (Protocol number 71559). Each participant signed an informed consent prior to the start of the experiment and all data were analyzed and reported anonymously.

### Pavlovian fear acquisition and reversal task

**Fig. 1:**
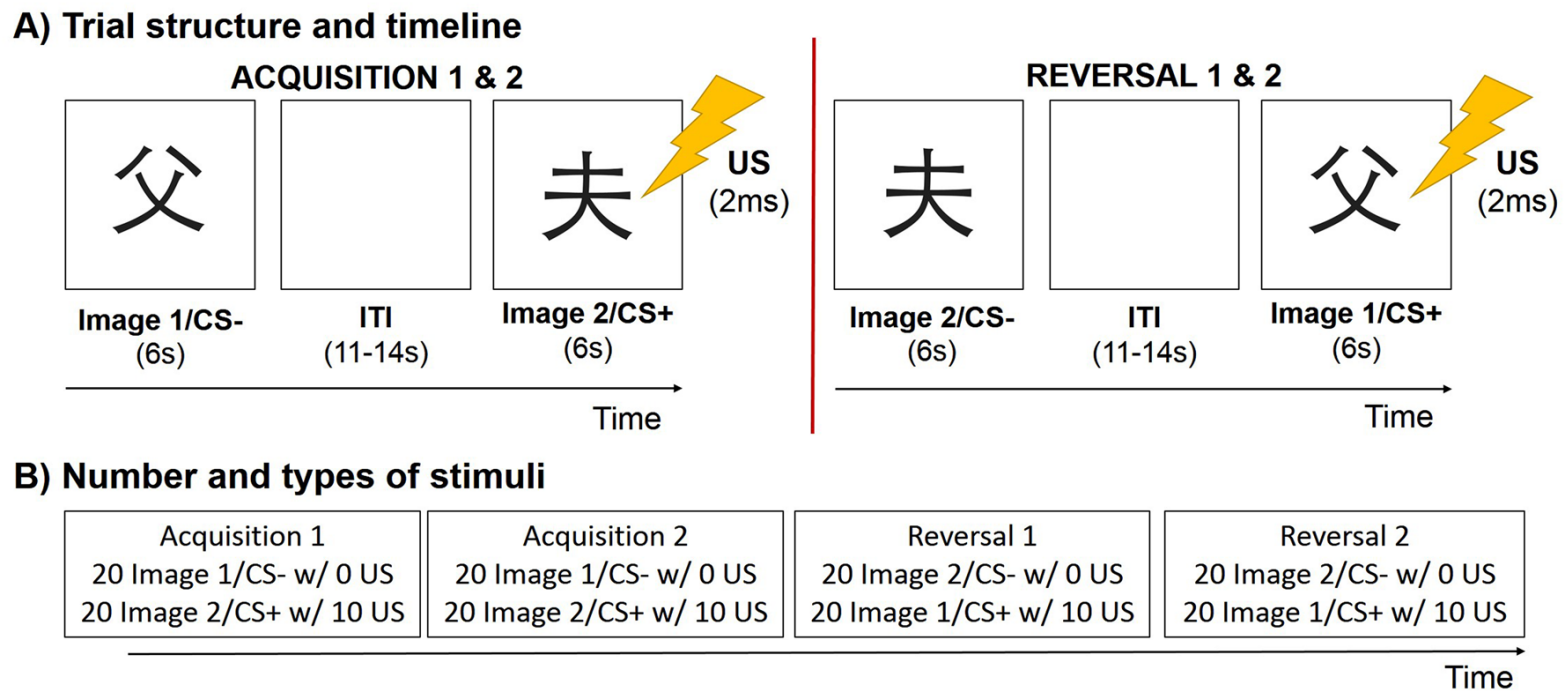
Pavlovian fear acquisition and reversal task. **A** Trial structure and timeline. Images were presented for 6 s, followed by a jittered 11-14 s intertrial interval. During acquisition and reversal, presentation of CS + (Image 1 in acquisition, Image 2 in reversal) co-terminated with an aversive electric shock unconditioned stimulus (US, of 2 ms duration). Note that during reversal, Image-US contingencies reversed relative to acquisition, such that the CS- corresponded to Image 2 and the CS + corresponded to the Image 1. **B** Number and types of stimuli presented during the task. Each acquisition and reversal block included 20 presentations of each Image. During each block, the CS + was reinforced with an aversive US on 10 trials (“w/ 10”, reinforcement rate of 50%), while the CS- was never paired with a US (“w/ 0”)

The experiment consists of two phases. During the first phase, the acquisition phase, participants view two different visual stimuli (Japanese hiragana) on screen (Starita and di Pellegrino 2018; Starita et al. 2019b). One hiragana, in the following denoted as Image 1, is used as a control stimulus (CS-), while the other hiragana, denoted as Image 2, acts as a conditioned stimulus (CS +). That is, following 50% of visualizations of the CS + stimulus, an aversive shock (unconditioned stimulus, US) was administered, whereas no shock ever occurred after the CS- stimulus. During the second phase, the reversal phase, the hiragana are reversed, so that Image 2 acts as the new CS- while Image 1 acts as the new CS + . The unconditioned stimulus (US) consists of a 2 ms electrostatic stimulation (Effting and Kindt 2007; Krypotos et al. 2014, 2015; Starita et al. 2022; Stemerding et al. 2022) administered in the right wrist (dominant hand) through a Digitimer stimulator (model DS7A, Digitimer Ltd., M = 51.25 mA, SD = 1.88 mA) via Ag/AgCl pregelatinized electrodes (Friendship Medical, SEAg-S-15000/15 × 20). The intensity of the shock is calibrated for each subject, via verbal feedback and an ascending level procedure, up to a level defined as 'very annoying and unpleasant, but never painful'. Figure 1 illustrates the experimental paradigm.

The task is generated thanks to the OpenSesame software (Mathôt et al. 2012). Each phase of the task (both acquisition and reversal) included two blocks (i.e., we have 4 blocks in total: Acq1, Acq2, Rev1 and Rev2), which were interspersed with a 5-min break. For each block, 40 stimuli were shown on screen (i.e., 40 trials, 20 per CS). Each stimulus was presented for 6 s, with an interstimulus time interval ranging from 11 to 14 s. In each block, stimuli followed each other in random order, with only constraints on the first two trials (one CS- and one CS + /US) and on the number of consecutive stimuli of the same type, never more than two.

Subjects received no information regarding which visual stimulus would be associated with the shock, only the following instructions at the beginning of each block were provided "You will see two different images, which will appear one at a time on the screen. Occasionally, the image may shock you. Your task is to figure out which image will shock you. Press any key to get started." The CS-US relationship is then learned from scratch with experience. During each block, the subjects' electroencephalographic signal and skin conductance were recorded.

### Skin conductance response (SCR)

Skin conductance was recorded during each block at 1000 Hz (10 Hz low-pass filter, gain switch set to 5) through the BIOPAC MP-150 system (Goleta, CA). Pregelatinized electrodes (BIOPAC EL501) were connected on the palmar surface of the left non-dominant, non-shocked hand. The signal was then digitized and down sampled at 200 Hz using Autonomate software (version 2.8, Green et al. 2014) to detect trough-to-peak SCR values. SCR was considered valid if trough-to-peak deflection began between 0.5 s and 4.5 s after Image onset, lasted no longer than 5 s, and was greater than 0.02 µS. Skin conductance values from trials that did not meet all of these criteria were set as zero SCR and retained in the analyses (Starita et al. 2019a).

Note that, in the present work, SCR is used for the sole purpose of demonstrating the correct acquisition and reversal of fear in our subject group. The relationship between SCR and brain rhythms (i.e., between the central nervous system and autonomic responses) was analyzed in the previous work. Similarly, subjective ratings of CS-US contingency awareness and CS valence collected at the end of each block confirmed acquisition and reversal at an explicit level. The details of such results can be found in (Starita et al. 2023).

### EEG recording and processing

EEG signals were collected from all participants through 63 wet Ag/AgCl electrodes. Each signal was referenced to the FCz and grounded to the FPz electrode. For all participants, the EEG signals were amplified by a BrainAmp DC amplifier (Brain Products, Gilching, Germany) and digitized at a sampling rate of 1000 Hz. Data corresponding to the four experimental blocks (Acq1, Acq2, Rev1 and Rev2) were then exported to MATLAB R2021a (MathWorks Inc., Natick MA, USA) and processed offline through custom-made scripts. A schematic illustration depicting the various processing steps is reported in the supplementary information (SI_1). Firstly, data were down-sampled at 500 Hz and processed with both a band-pass filter (1–60 Hz) and a notch filter (50 Hz) to remove the irrelevant EEG spectral content and electric coupling interferences. Specifically, with regard to the band-pass filter, we implemented a 5th-order elliptic filter, with a passband ripple of 0.1 dB and a stopband attenuation of 40 dB, and performed zero-phase digital filtering. With these specifications, the frequency contents in the bands of interest (from 4 Hz for the theta rhythm to 42 Hz for the gamma rhythm) are not compromised. Then, 40 stimulus-locked epochs from 0 (stimulus onset) to 6 s (stimulus duration) were extracted from EEG recordings of each block, corresponding to 20 Image 1 trials and 20 Image 2 trials. Moreover, the 10 s preceding the onset of the first stimulus were extracted from Acq1 and defined as the participant’s baseline signal. At this stage, the different epochs were visually inspected, and no trials were deemed necessary to be discarded. Once concatenated the extracted signals along the time dimension (baseline, Acq1, Acq2, Rev1 and Rev2), bad channels were identified by computing the correlation coefficient between each electrode and the others. More precisely, for each EEG electrode we calculated the mean value of the four highest (absolute) correlations and marked as bad channels those electrodes whose mean value was < 0.4 (Bigdely-Shamlo et al. 2015; da Cruz et al. 2018). Then, the remaining good channels were re-referenced to the average of all electrodes, and the reference electrode (FCz) recovered.

Subsequently, to remove the artefactual components from EEG data, we performed the Independent Component Analysis (ICA) using the EEGLAB MATLAB toolbox (https://sccn.ucsd.edu/eeglab/index.php). Independent Components (ICs) containing artifacts were at first identified through an EEGLAB plugin named ‘IClabel’, which defines the probability of each extracted IC to be a brain-driven (‘Brain’) or a non-brain-driven activity (‘Muscle’, ‘Eye’, ‘Heart’, ‘Line Noise’, ‘Channel Noise’ and ‘Other’). After rejecting the ICs classified as ‘Brain’ with less than 5% probability, we visually inspected all the remaining components (scalp map, time and spectral activity) and further removed only those showing clear artifactual activity. Finally, artifact-cleaned EEG signals were used to retrieve the previously identified bad channels using the spherical interpolation, and the 64 EEG signals were again re-referenced to the average of all electrodes.

### Cortical sources reconstruction

Cortical source activity was reconstructed starting from the 64 artifact-cleaned EEG signals. The estimation of intracortical current densities was performed using the method eLORETA (exact Low Resolution Electromagnetic Tomography, LORETA-KEY©® software package, version: v20200414), a functional imaging technique belonging to the family of linear inverse solutions for 3D EEG source distribution modeling. Precisely, the algorithm computes the weighted minimum norm solution, so that the particular weights used in this solution endow eLORETA with the property of exact localization of test point sources under ideal (noise free) conditions (Pascual-Marqui et al. 2011).

The software employs a template three-layers head model (MNI152 template) comprising the scalp, the outer skull surface, and the inner skull surface and registered to the Talairach human brain atlas. The solution space is restricted to the grey matter of the reference brain, divided into 6239 voxels at 5 mm spatial resolution. The software LORETA-KEY©® was exploited to compute the inversion matrix starting from the Talairach coordinates of the 64 electrodes, while all subsequent processing steps were implemented in MATLAB.

Since each cortical source is described by a three-dimensional current density vector, by right multiplying the inversion matrix by the 64 EEG signals, we can extract the three scalar components of the current density vector for the 6239 voxels and at each time instant. Then, as the choice of constrained dipole orientations was made, the 3D current densities were projected on the voxels’ normal versor obtaining one time series for voxel.

Finally, according to the atlas used by LORETA-KEY©® (76 ROIs, see Table 1) voxels were grouped in functionally significant Regions of Interest (ROIs), and the signal representing each ROI was obtained as the mean activity of the voxels belonging to the ROI. The relationship between the ROIs’ names, MNI coordinates, and Brodmann areas is provided in the supplementary information (SI_2).

**Table 1:** List of the regions of interest (ROIs) in which the cerebral cortex has been divided

Angular gyrus (AG)	Lingual gyrus (LG) *	Precuneus (PCU) *
Anterior cingulate (AC) *	Medial frontal gyrus (MeFG) *	Rectal gyrus (RG)
Cingulate gyrus (CG) *	Middle frontal gyrus (MFG)	Sub-gyral (SG)
Cuneus (CU) *	Middle occipital gyrus (MOG)	Subcallosal gyrus (SCG) *
Extra-nuclear (EN)	Middle temporal gyrus (MTG)	Superior frontal gyrus (SFG) *
Fusiform gyrus (FG)	Orbital gyrus (OG)	Superior occipital gyrus (SOG)
Inferior frontal gyrus (IFG)	Paracentral lobule (PCL) *	Superior parietal lobule (SPL)
Inferior occipital gyrus (IOG)	Parahippocampal gyrus (PHG)	Superior temporal gyrus (STG)
Inferior parietal lobule (IPL)	Postcentral gyrus (PCG)	Supramarginal gyrus (SMG)
Inferior temporal gyrus (ITG)	Posterior cingulate (PC) *	Transverse temporal gyrus (STG)
Insula (IN)	Precentral gyrus (PG)	Uncus (UN)

Please note that each area includes a left, right and in some cases (10 out of 33, indicated by asterisks) a medial portion, thus totally resulting in 76 regions. Abbreviations used in the article are shown in parentheses

### Cortical power computation

The power spectral density (PSD) was evaluated on the 76 reconstructed cortical ROIs and during each trial using the Welch’s periodogram method (Hamming window of 2 s, 50% overlap, 10 s zero padding).

Power analysis in theta (4–8 Hz), alpha (8–14 Hz) and gamma (30–42 Hz) bands was then performed separately in each experimental block (Acq1, Acq2, Rev1 and Rev2) and for each stimulus (Image 1 and Image 2). In particular, for each participant, the following analyses were performed in each ROI. The power was computed in each frequency band and each trial, and normalized to the baseline condition. That is, for each participant the power in the alpha, theta and gamma bands, for each trial and each phase, was divided by the power of the respective band computed in the 10 s participant's baseline. This trial-by-trial power signal was then separated between Image 1 and Image 2 (20 trials per image and per block) and used for two computations. 1) For each frequency band and each block, a mean power was computed by averaging the trial-by-trial power over the 20 trials of each image, to obtain one power value for block and stimulus. 2) For each frequency band and each block, the trial-by-trial power was used to compute a moving average signal for each image, considering a window of 3 trials (sliding one trial at a time), in order to evaluate power temporal evolution for each stimulus over the four experimental blocks. Since the chosen window is odd, it is centered on the trial in the current position. When there are not enough trials to fill the window (that is, regarding the first and last trials of each block), the window size is automatically truncated and the average is taken over only the two trials that fill the window.

Importantly, the data resulting from mean power computation (computation 1) were used to identify, without any a priori assumption, the ROIs and rhythms implicated in fear conditioning, and thus deserving an in-depth inspection in this work. To this end, for each frequency band we followed two steps. Step 1: we selected all areas that exhibit a statistical significant difference (corrected) in power between Image 1 and Image 2 (i.e., between CS + and CS-) in at least one of the four experimental blocks, to detect a possible involvement either in acquisition or reversal. We anticipate here that all these corrected significances occur only in the acquisition phase. Step 2: since the focus in this work is on reversal, we further restricted our analysis to those ROIs (among those selected in step 1) that exhibit a statistically significant difference (although not corrected) between Image 1 and Image 2 both during acquisition and reversal. As shown in “Results”, this resulted in two regions for the theta rhythm, eleven regions for the alpha rhythm, and no region for the gamma rhythm.

### Functional connectivity through frequency-domain Granger causality

To further investigate the possible neural mechanisms underlying fear acquisition and reversal, we evaluated the functional connectivity in each considered frequency band among the 76 reconstructed cortical ROIs by using the Spectral Granger Causality estimator, which provides weighted and directional metrics of the causal interactions between ROIs. The connectivity analysis was limited to the theta and alpha bands, since the power analysis in the gamma band provided inconclusive results (see “Results”). The Granger Causality is based on the autoregressive (AR) modeling framework and estimates the functional connectivity between ROIs by comparing the prediction ability of two AR models (of a certain order $p$) on the same process ${x}_{k,j}$. Specifically, given two time series ${x}_{k,i}[n]$ and ${x}_{k,j}[n]$, where *n* is the discrete time ($n=0, 1,\dots ,N-1$), representing the activity at two distinct cortical ROIs (${ROI}_{i}$ and ${ROI}_{j}$) for each participant $k$ ($k=1,\dots ,19$). The Granger Causality estimator quantifies the causal interaction from ${ROI}_{i}$ to ${ROI}_{j}$ as the improvement in predictability of ${x}_{k,j}[n]$ when using a bivariate AR model, based on both past values of ${x}_{k,j}$ and past values of ${x}_{k,i}$, compared to a univariate AR model, based only on past values of ${x}_{k,j}$.

Frequency-domain Granger Causality can be formalized starting from the spectral derivation of the bivariate representation of the activity of the two ROIs, ${x}_{k,j}[n]$ and ${x}_{k,i}[n]$ via the Fourier Transformation. According to Geweke (1982, 1984) the power spectrum of a time series ${x}_{k,j}[n]$ can be decomposed into an ‘intrinsic’ and a ‘causal’ part, considering the latter predicted by the other time series ${x}_{k,i}[n]$.

The GC spectrum from $i$ to $j$ ${(GC}_{i\to j}(f))$ is defined as the logarithm of the ratio between the total power spectrum of ${x}_{k,j}[n]$ at frequency $f$ and the difference between the total power spectrum and the ‘causal’ power predicted by ${x}_{k,i}[n]$ at the same frequency. Accordingly, at a given frequency *f,* the estimated quantity ${GC}_{i\to j}\left(f\right)$ is zero when the causal power of ${x}_{k,i}[n]$ onto ${x}_{k,j}[n]$ is zero and increases (> 0) as the causal power increases. For each participant $k$, a GC spectrum was computed in each block and for each stimulus by linking 20 trials for each experimental block and stimulus. In all cases, the order $p$ of the AR models was set equal to 30 on the basis of a previous analysis (Magosso et al. 2021; Tarasi et al. 2021) which showed that for $p\ge 30$ the estimated values of GC do not change substantially. It is worth noting that spectral GC provides a connectivity matrix ($GC\left(f\right):$ 76 × 75, discarding auto connectivity) for each frequency sample ($n\,\, sample = 2501, frequency\,\, resolution=0.1 Hz$). To obtain a single connectivity value representative of the rhythms under analysis, we computed the mean value of GC(f) in the given frequency band. Additionally, for each participant, the theta and alpha connectivity matrices were normalized so that the sum of all connections in each matrix is equal to 100. Then, from these normalized matrices (named *complete* connectivity matrices), *sparse* normalized connectivity matrices were obtained by performing a statistical analysis between the two types of stimuli (Image 1 and Image 2) independently for each experimental block, using the non-parametric permutation *t*-test (see details below in the “Statistical analysis”). Hence, for each block and for each frequency band, only connections significantly different (*p* value < 0.05) between Image 1 and Image 2 among all the 76 × 75 possible connections were retained in each subject connectivity matrix, while the others were set to zero.

Finally, both the complete and the resulting sparse connectivity matrices were averaged across participants to characterize each block and each stimulus (4 blocks × 2 stimuli) with a connectivity matrix.

To understand how power changes are transmitted from a generative node to others nodes in the network, we focused on the set of connections exiting from the areas identified in the previous frequency domain analysis, following the two-step method described in ‘Cortical Power computation’. The resulting functional network is represented through a graph where the involved cortical ROIs are the nodes and the connectivity values are displayed by weighted and directed arrows.

### Statistical analysis

For each experimental block, a two-tailed permutation-based t-test for dependent samples between Image 1 and Image 2 was performed on SCR first, and then on the normalized mean power and connectivity data in each considered frequency band. The test was performed through a custom-made MATLAB script from the functions implemented in the FieldTrip toolbox (Oostenveld et al. 2010). It should be noted that the two-tailed test was used as we had no a priori hypothesis about how the power and connectivity were varying (increase/decrease) between the two stimuli. The distribution of the t-statistic for each cortical ROI under the null hypothesis was empirically realized by generating 5000 random permutations of the observed values between the 2 stimulus conditions (Monte Carlo method). The uncorrected p-value was the proportion of the permutation distribution greater than or at most equal to the observed t-statistic computed on the non-permuted values. Then, for cortical mean power analysis (i.e. average power over the 20 trials) a correction for multiple comparisons (76 comparisons, one per ROI) was achieved, separately for each block, using the false discovery rate correction (Benjamini–Hochberg procedure) (Benjamini and Hochberg 1995). Also, the SCR statistics largely survive correction (4 comparisons, one per block).

Conversely, no correction was performed for connectivity analysis, since we did not follow a confirmatory (i.e., hypothesis testing) approach but rather an exploratory one (hypothesis generation), not knowing a priori where connections might show significant differences between Image 1 and Image 2. Therefore, in this case, a *p* value < 0.05 does not indicate whether the null hypothesis is supported but rather represents a measure of how statistically convincing the difference in connectivity is compared to the others.

Finally, for scalp-level and moving average analyses, a cluster-based permutation t-test (Maris and Oostenveld 2007) was performed, to account for spatial dependence between electrodes and temporal dependence between trials, respectively.

## Results

### Skin conductance response analysis

First, a statistical analysis was performed between the SCR during the presentation of Image 1 and Image 2 in each block, to assess the correct acquisition and reversal of fear.

For each block (Acq1, Acq2, Rev1 and Rev2), we found a statistically significant difference between images in SCR values. In detail: p_Acq1 = $3.9*{10}^{-4}$, p_Acq2 = $3.9*{ 10}^{-4}$, p_Rev1 = $6.0*{ 10}^{-3}$, and p_Rev2= $3.9*{ 10}^{-4}$ (uncorrected). Moreover, in all blocks SCR was higher during CS + than CS- (i.e., during the presentation of Image 2 in Acq1 and Acq2, and Image 1 in Rev1 and Rev2). This analysis confirms the successful acquisition and reversal of fear for the subject group. A figure showing the group means SCR values for the two images, in the four blocks, is provided in the supplementary information (SI_3).

### Scalp-level EEG power analysis

**Fig. 2:**
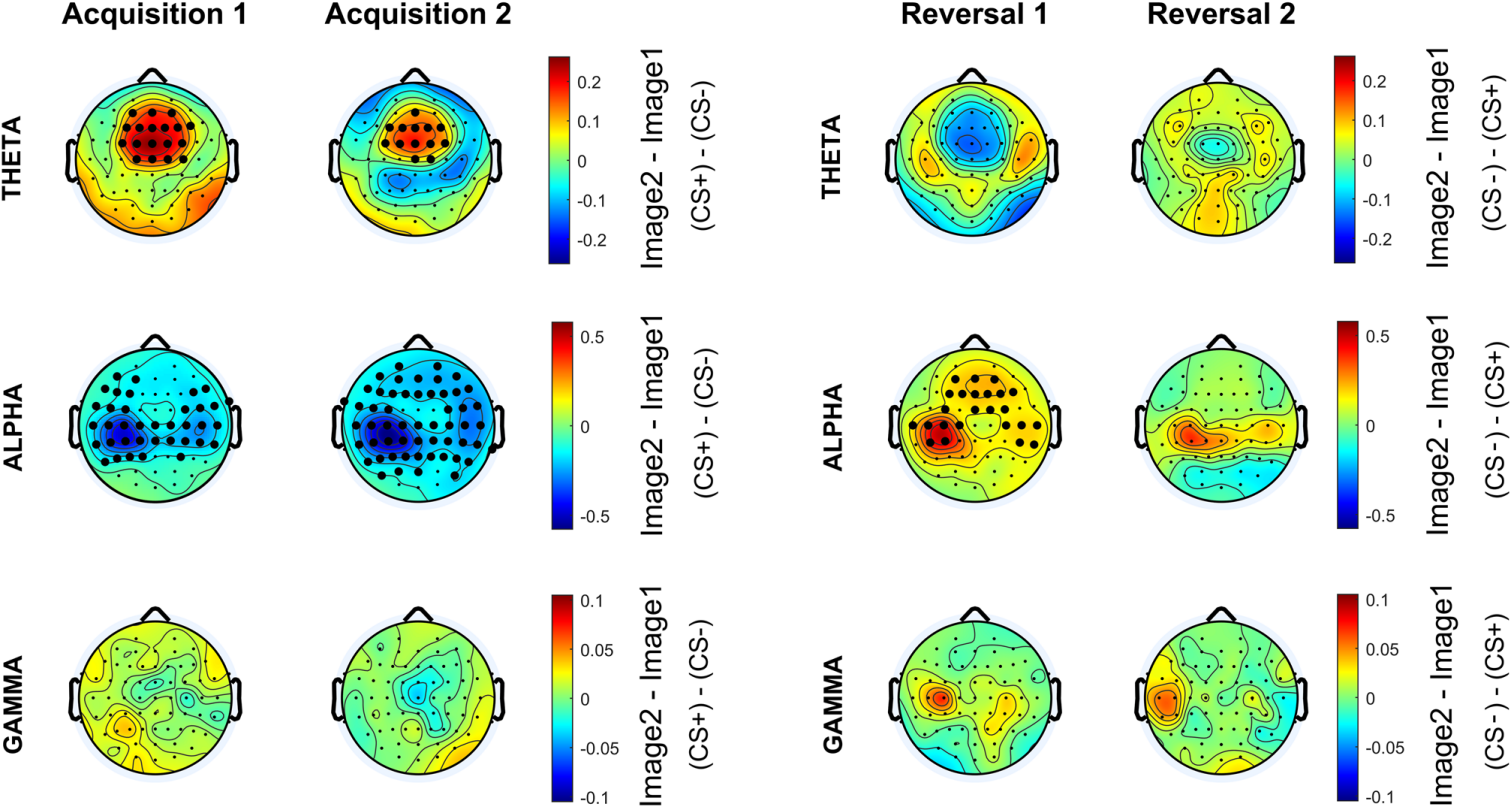
Differential (Image2-Image1) scalp-level maps of the normalized mean power in the theta, alpha and gamma bands, in the four blocks. Negative values (colors tending toward blue) indicate higher power during the visualization of Image 1 (CS- in acquisition, CS + in reversal), while positive values (colors tending toward red) indicate higher power during the visualization of Image 2 (CS + in acquisition, CS- in reversal). Channels bolded in black indicate the presence of corrected statistical significance (*p* < 0.05, cluster-based correction). In fronto-medial channels, theta power is always greater during CS + (Image 2 in Acq1 and Acq2; Image 1 in Rev1 and Rev2) than CS-. In left parietal channels, alpha power is always greater during CS- (Image 1 in Acq1 and Acq2; Image 2 in Rev1 and Rev2) than CS + . Gamma power shows a less consistent trend among the four blocks

This work aims to investigate the role of brain rhythms at the cortical level. However, to ensure the reliability of our findings, strengthen their value, and enable comparisons with previous studies (e.g., Mueller et al. 2014; Sperl et al. 2019; Bierwirth et al. 2021), scalp-level power was also computed for a preliminary analysis. Differential scalp-level maps for the theta, alpha, and gamma bands normalized mean power are reported in Fig. 2. Theta band shows greater power during CS + (i.e., Image 2 in Acq1 and Acq2; Image 1 in Rev1 and Rev2) than CS- in fronto-medial channels, for all the four blocks; alpha band shows greater power during CS- (i.e., Image 1 in Acq1 and Acq2; Image 2 in Rev1 and Rev2) than CS + in left parietal channels, for all the four blocks; while gamma band power shows less consistency among all phases of the experiment. Cluster-based analysis shows statistically significant (corrected) differences especially in the acquisition phase.

Data resulting from the mean power analysis (computation 1, see “Cortical Power Computation”) were used to identify, without any a priori assumptions, the ROIs and rhythms most involved in fear acquisition and reversal. For these regions, we displayed the power mean value across the four blocks and the moving average of the trials. Finally, we further performed functional connectivity analysis.

### Cortical sources power analysis

#### Theta power

**Fig. 3:**
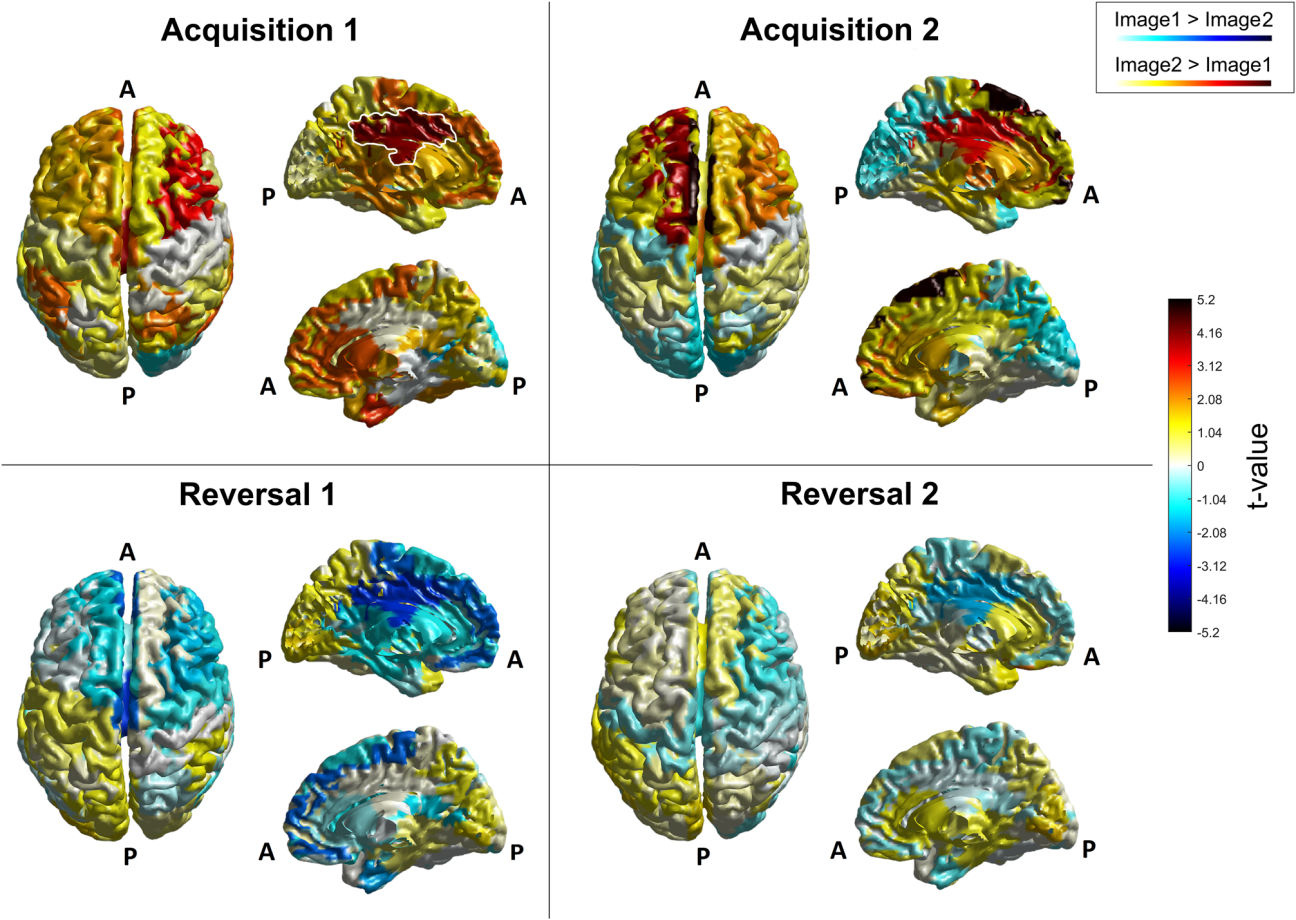
Student's ‘*t*’ values, resulting from the statistical comparison between the two images, carried out on theta mean power, in the four blocks and over all the 76 cortical ROIs. In each panel, the left column represents the top view of the cerebral cortex while the right column represents the medial left (top) and the medial right (bottom) view of the cerebral cortex. White outline in the 'Acquisition 1' panel is used to highlight areas that have been selected for further analysis. That is, for the theta rhythm, the left and medial cingulate gyrus (CG l and CG m, visible in the left medial view). Letter ‘A’ stands for ‘Anterior’, letter ‘P’ for ‘Posterior’. The color bar corresponds to uncorrected *t*-values. Positive values (colors tending toward red) indicate higher power during the visualization of Image 2 (CS + in acquisition, CS- in reversal), while negative values (colors tending toward blue) indicate higher power during the visualization of Image 1 (CS- in acquisition, CS + in reversal)

To get a global view, Fig. 3 shows the Student's ‘t’ values resulting from the 76 ROI-wise statistical comparison between the two images, carried out on theta mean power. All the four blocks and all the 76 cortical ROIs are depicted. This figure shows that strong theta differences are especially evident in a portion of the cortex close to the left cingulate gyrus. Furthermore, the power is greater for Image 2 (i.e., CS +) than Image 1 (i.e. CS-) during the acquisition phase, and is inverted (i.e., is greater for Image 1, the new CS +) passing from the acquisition to the reversal phase: i.e., theta power is greater during CS + than CS- both during acquisition and reversal. It is important to note that, as seen in Fig. 3, several regions show high absolute ‘t’ values (i.e., both positive and negative), but without reaching the statistical level that survives correction. For this reason, these regions are not considered for the subsequent analyses, but may be of interest for future investigation. A figure similar to Fig. 3, depicting only the significant (corrected) regions for the theta power, is shown in the supplementary information (SI_4).

To better summarize the results, Table 2 lists all regions which exhibit a statistically significant difference (corrected) in theta power between Image 1 and Image 2 in at least one block of the experiment (step 1 in Methods section). These are the left cingulate gyrus (CG l), the left superior frontal gyrus (SFG l), the medial cingulate gyrus (CG m), and the medial superior frontal gyrus (SFG m). All these corrected statistical differences are evident during the acquisition phase but not during the reversal phase. Moreover, according to the second criterion delineated in the Methods section (step 2), two of the previous regions [i.e., the left cingulate gyrus (CG l) and the medial cingulate gyrus (CG m)] exhibit a significant (but uncorrected) statistical difference in the first reversal block, revealing that these regions are implicated not only in acquisition but likely also in reversal. In the following, we will focus the attention on these two regions to better characterize the theta band.

**Table 2:** Names and block-by-block corrected *p* values of the ROIs that show significant corrected statistical differences in normalized theta power between CS + and CS − in at least one block

ROIs	Acquisition 1	Acquisition 2	Reversal 1	Reversal 2
Left cingulate gyrus (CG l)	*p* = 0.046 (0.0012)	*p* = 0.010 (3.9*10^^−4^)	NS (*p* = 0.029)	NS (NS)
Left superior frontal gyrus (SFG l)	NS (NS)	*p* = 0.010 (3.9*10^^−4^)	NS (NS)	NS (NS)
Medial cingulate gyrus (CG m)	*p* = 0.030 (3.9*10^^−4^)	NS (*p* = 0.021)	NS (*p* = 0.0068)	NS (NS)
Medial superior frontal gyrus (SFG m)	NS (NS)	*p* = 0.010 (3.9*10^^−4^)	NS (NS)	NS (NS)

The uncorrected p-values are shown between brackets. NS signifies that no statistically significant difference was observed

**Fig. 4:**
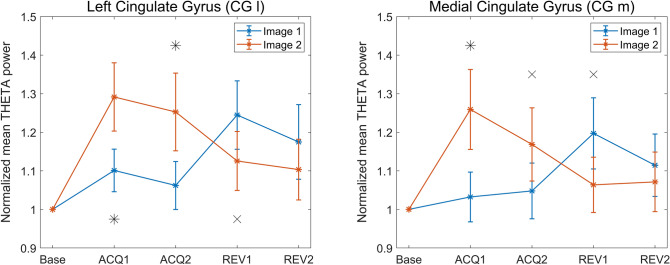
Normalized mean power in the theta band, for all four blocks and both images, in the left cingulate gyrus (CG l, left column) and in the medial cingulate gyrus (CG m, right column). The power for Image 1 is depicted in blue and the power for Image 2 in red, accompanied in each block by the respective SEM bar. Asterisks indicate presence of corrected statistical significance (*p* < 0.05, false discovery rate corrected) in that particular block while crosses denote the presence of a statistical significance (*p* < 0.05, uncorrected) which does not survive correction for multiple comparisons. It is well evident the power inversion in passing from acquisition to reversal, i.e., theta power is always greater during CS + (Image 2 in Acq1 and Acq2; Image 1 in Rev1 and Rev2) than CS-

Figure 4 shows the block-by-block normalized theta band power of CG l and CG m. It is noticeable that theta power is greater for the CS + stimulus both during acquisition and reversal (Image 2 in Acq1 and Acq2, Image 1 in Rev1 and Rev2). In agreement with Fig. 3, this difference is mostly evident in acquisition and is reduced during reversal. Especially in the second reversal block, the power difference is statistically insignificant even when uncorrected.

**Fig. 5:**
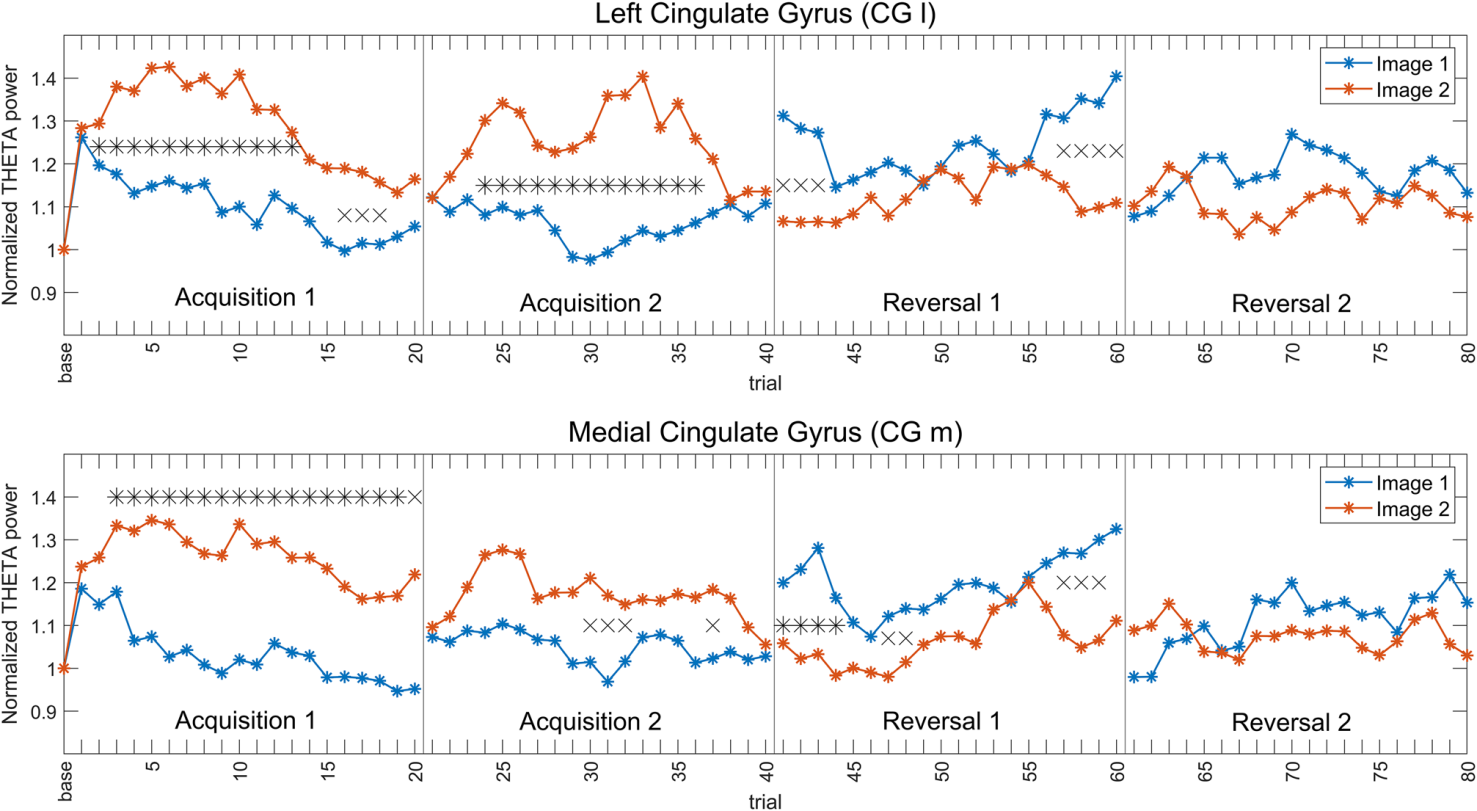
Moving averages (w = 3 trials) of normalized theta power, trial by trial, for the four blocks. The top row shows the normalized power of the left cingulate gyrus (CG l), the bottom row the normalized power of the medial cingulate gyrus (CG m). The two images are shown in the same color (blue for Image 1, red for Image 2). Asterisks indicate the presence of corrected statistical significance (*p* < 0.05, cluster-based correction) for that particular trial, while crosses indicate the presence of uncorrected statistical significance (*p* < 0.05). Vertical lines are used to delineate the four different blocks. It is evident that theta power is always greater during CS + (Image 2 in Acq1 and Acq2; Image 1 in Rev1 and Rev2), and that inversion occurs already after the first reversal trial of the moving average

Figure 5 shows the trial by trial moving average (window length = 3 trials) of theta power in the two selected regions, CG l and CG m. The figure confirms that theta-band power is greater for the CS + than for the CS- in almost all trials (Image 2 in Acq1 and Acq2, Image 1 in Rev1 and Rev2). The inversion in reversal 1 occurs very quickly, being already evident after the first trial of the moving average. The greatest power difference between the two stimuli is observed in the two acquisition blocks, while in reversal this difference progressively decreases. Indeed, in reversal 2, the power difference between the two images is less marked, as confirmed by the statistical analysis, which does not show any statistically significant difference in any trial of this block. This appears as a result of the drastic fall in theta power during Image 1 (new CS +) at the beginning of the reversal 2.

#### Alpha power

**Fig. 6:**
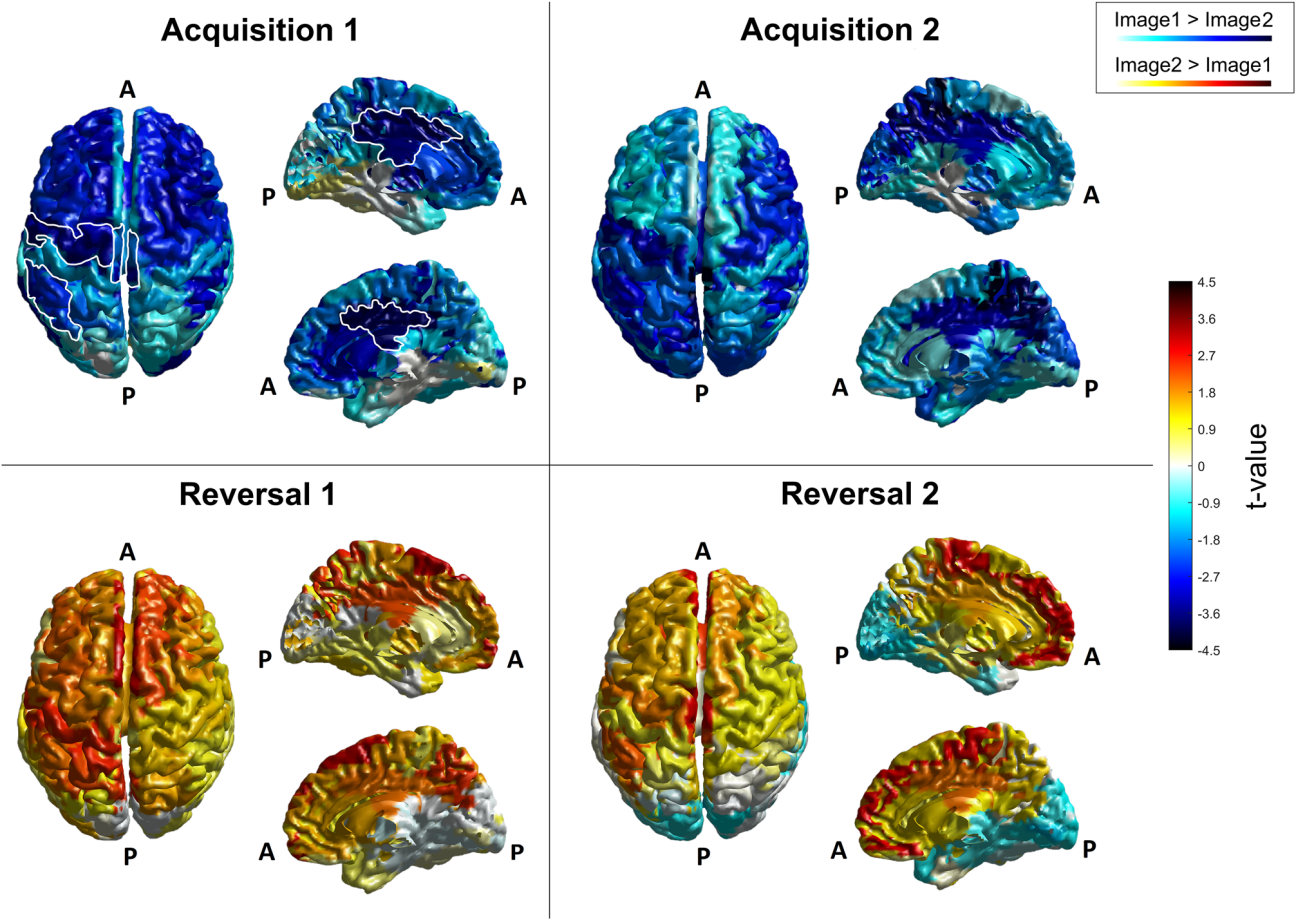
Student's ‘t’ values, resulting from the statistical comparison between the two images, carried out on alpha mean power, in the four blocks and over all the 76 cortical ROIs. In each panel, the left column represents the top view of the cerebral cortex while the right column represents the medial left (top) and the medial right (bottom) view of the cerebral cortex. White outlines in the 'Acquisition 1' panel are used to highlight areas that have been selected for further analysis. That is, for the alpha rhythm, the left inferior parietal lobule, the left and medial paracentral lobule, the left precentral gyrus (IPL l, PCL l, PCL m and PG l, visible in the top view) and the left and right cingulate gyrus (CG l and CG r, visible in the left and right medial view). Letter ‘A’ stands for ‘Anterior’, letter ‘P’ for ‘Posterior’. The color bar corresponds to uncorrected *t*-values. Negative values (colors tending toward blue) indicate higher power during the visualization of Image 1 (CS- in acquisition, CS + in reversal), while positive values (colors tending toward red) indicate higher power during the visualization of Image 2 (CS + in acquisition, CS- in reversal)

Figure 6 shows the Student's ‘t’ values resulting from the 76 ROI-wise statistical comparison between the two images, carried out on alpha mean power. All the four blocks and all the 76 cortical ROIs are depicted. This figure shows that significant alpha differences are evident in a large portion of the parietal cortex in the left hemisphere (i.e., contralateral to the shocked hand) and these changes are also evident (although less marked) during reversal. Alpha power is smaller for Image 2 (i.e., CS +) during the acquisition phase, and becomes smaller for Image 1 (i.e. the new CS +) during reversal. Also in this case, other areas exhibit stronger statistical differences which however do not survive correction. As well as for theta power, a figure similar to Fig. 6, showing only the significant (corrected) regions for the alpha power, is provided in the supplementary information (SI_4).

Table 3 shows all regions which exhibit a significant statistical difference (corrected) in alpha power between Image 1 and Image 2 in at least one block of the experiment (step 1 in Methods section). These differences occur during acquisition only. Moreover, according to the second criterion (step 2), among these, eleven areas show at least one statistical significance (uncorrected) in the reversal phase of the experiment.

**Table 3:** Names and block-by-block corrected p-values of the ROIs that show significant differences between normalized alpha power between CS + and CS − in at least one block

ROIs	Acquisition 1	Acquisition 2	Reversal 1	Reversal 2
Right anterior cingulate (AC r)	*p* = 0.30 (0.060)	NS (NS)	NS (NS)	NS (NS)
Right cingulate gyrus (CG r)	*p* = 0.023 (0.0012)	*p* = 0.037 (0.0072)	NS (NS)	(*p* = 0.031)
Right extra-nuclear (EN r)	*p* = 0.046 (0.012)	NS (NS)	NS (NS)	NS (NS)
Right inferior frontal gyrus (IFG r)	(*p* = 0.042)	*p* = 0.035 (0.0040)	NS (NS)	NS (NS)
Right inferior parietal lobule (IPL r)	*p* = 0.043 (0.010)	*p* = 0.030 (0.0024)	NS (NS)	NS (NS)
Right middle frontal gyrus (MFG r)	*p* = 0.024 (0.0020)	(*p* = 0.029)	NS (NS)	NS (NS)
Right middle occipital gyrus (MOGr)	*p* = 0.024 (0.0032)	(*p* = 0.035)	NS (NS)	NS (NS)
Right posterior cingulate (PC r)	*p* = 0.043 (0.011)	*p* = 0.035 (0.0060)	NS (NS)	NS (NS)
Right superior frontal gyrus (SFG r)	*p* = 0.043 (0.0010)	NS (NS)	(*p* = 0.021)	NS (NS)
Right transverse temporal gyrus (TTG r)	*p* = 0.030 (0.0064)	(*p* = 0.046)	NS (NS)	NS (NS)
Left angular gyrus (AG l)	NS (NS)	*p* = 0.044 (0.0092)	NS (NS)	NS (NS)
Left cingulate gyrus (CG l)	*p* = 0.024 (0.0020)	*p* = 0.035 (0.0052)	(*p* = 0.026)	NS (NS)
Left extra-nuclear (EN l)	*p* = 0.023 (0.0012)	(*p* = 0.014)	(*p* = 0.016)	NS (NS)
Left inferior parietal lobule (IPL l)	(*p* = 0.020)	*p* = 0.035 (0.0060)	(*p* = 0.022)	(*p* = 0.022)
Left insula (IN l)	*p* = 0.024 (0.024)	NS (NS)	NS (NS)	NS (NS)
Left medial frontal gyrus (MeFG l)	*p* = 0.023 (8.0*10^^−4^)	NS (NS)	NS (NS)	(*p* = 0.0028)
Left middle occipital gyrus (MOG l)	NS (NS)	*p* = 0.010 (3.9*10^^−4^)	NS (NS)	NS (NS)
Left middle temporal gyrus (MTG l)	*p* = 0.024 (0.0032)	*p* = 0.035 (0.0060)	NS (NS)	NS (NS)
Left paracentral lobule (PCL l)	*p* = 0.024 (0.0032)	*p* = 0.024 (0.0016)	NS (NS)	(*p* = 0.047)
Left precentral gyrus (PG l)	*p* = 0.023 (7.9*10^^−4^)	*p* = 0.010 (3.9*10^^−4^)	(*p* = 0.038)	(*p* = 0.048)
Left precuneus (PCU l)	NS (NS)	*p* = 0.035 (0.0064)	NS (NS)	NS (NS)
Left superior frontal gyrus (SFG l)	*p* = 0.030 (0.0060)	NS (NS)	NS (NS)	NS (NS)
Left supramarginal gyrus (SMG l)	*p* = 0.028 (0.0044)	(*p* = 0.015)	NS (NS)	NS (NS)
Left transverse temporal gyrus (TTG l)	*p* = 0.028 (0.0040)	*p* = 0.035 (0.0044)	NS (NS)	NS (NS)
Medial anterior cingulate (AC m)	*p* = 0.046 (0.013)	(*p* = 0.036)	NS (NS)	(*p* = 0.023)
Medial cingulate gyrus (CG m)	NS (NS)	*p* = 0.030 (0.0028)	NS (NS)	NS (NS)
Medial paracentral lobule (PCL m)	*p* = 0.030 (0.0060)	*p* = 0.023 (0.0012)	NS (NS)	(*p* = 0.022)
Medial precuneus (PCU m)	NS (NS)	*p* = 0.010 (3.9*10^^−4^)	(*p* = 0.024)	NS (NS)

The uncorrected *p* values are shown between brackets. NS signifies that no statistically significant difference was observed

For the sake of brevity, in the following figures, we will focus attention on six regions [right cingulate gyrus (CG l), left cingulate gyrus (CG r), left inferior parietal lobule (IPL l), left precentral gyrus (PG l), left and medial paracentral lobules (PCL l and PCL m)], which show a clear power difference between images, a clear inversion during reversal, and are limbic, motor or somatosensory areas already reported in the literature as belonging to the so-called "Fear Network" (Tovote et al. 2015; Fullana et al. 2016; Lai 2019; Hudson et al. 2020). Normalized alpha-power differences in the remaining five regions [right superior frontal gyrus (SFG r), left extra-nuclear (EN l), left medial frontal gyrus (MeFG l), medial anterior cingulate (AC m) and medial precuneus (PCU m)], which nonetheless show similar trends, are reported for completeness in the supplementary information (SI_5).

**Fig. 7:**
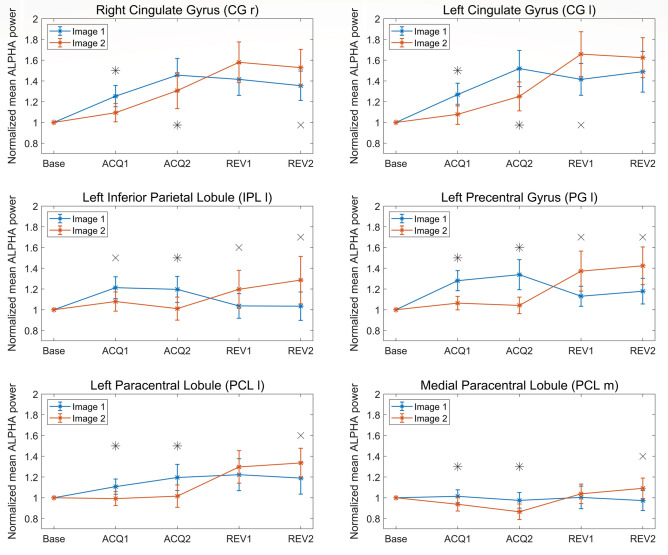
Normalized mean power in the alpha band, for all four blocks and both images, in the right and left cingulate gyrus (CG r and CG l, top row), left inferior parietal lobule (IPL l, middle row, left column), left precentral gyrus (PG l, middle row, right column), left and medial paracentral lobule (PCL l and PCL m, bottom row). Results for Image 1 are depicted in blue and those for Image 2 in red, accompanied in each block by the respective SEM bar. Asterisks indicate the presence of corrected statistical significance (*p* < 0.05, false discovery rate correction) in the specific block, while crosses denote the presence of a statistical significance (*p* < 0.05, uncorrected) which does not survive correction for multiple comparisons. It is well evident the power inversion in passing from acquisition to reversal, i.e., alpha power is always greater during CS- (Image 1 in Acq1 and Acq2; Image 2 in Rev1 and Rev2) than CS +

Figure 7 shows the block-by-block patterns of alpha power for the six selected regions. It is evident that the alpha power is higher in the CS- (i.e., during the presentation of Image 1 in Acq1 and Acq2, Image 2 in Rev1 and Rev2) than in CS + . This difference is especially evident and statistically significant during the acquisition phase but is also present during the reversal phase (although with reduced statistical significance due to the greater inter-subject variability). In addition, the alpha power in the CG r and the CG l shows the tendency to increase for both stimuli, block by block.

**Fig. 8:**
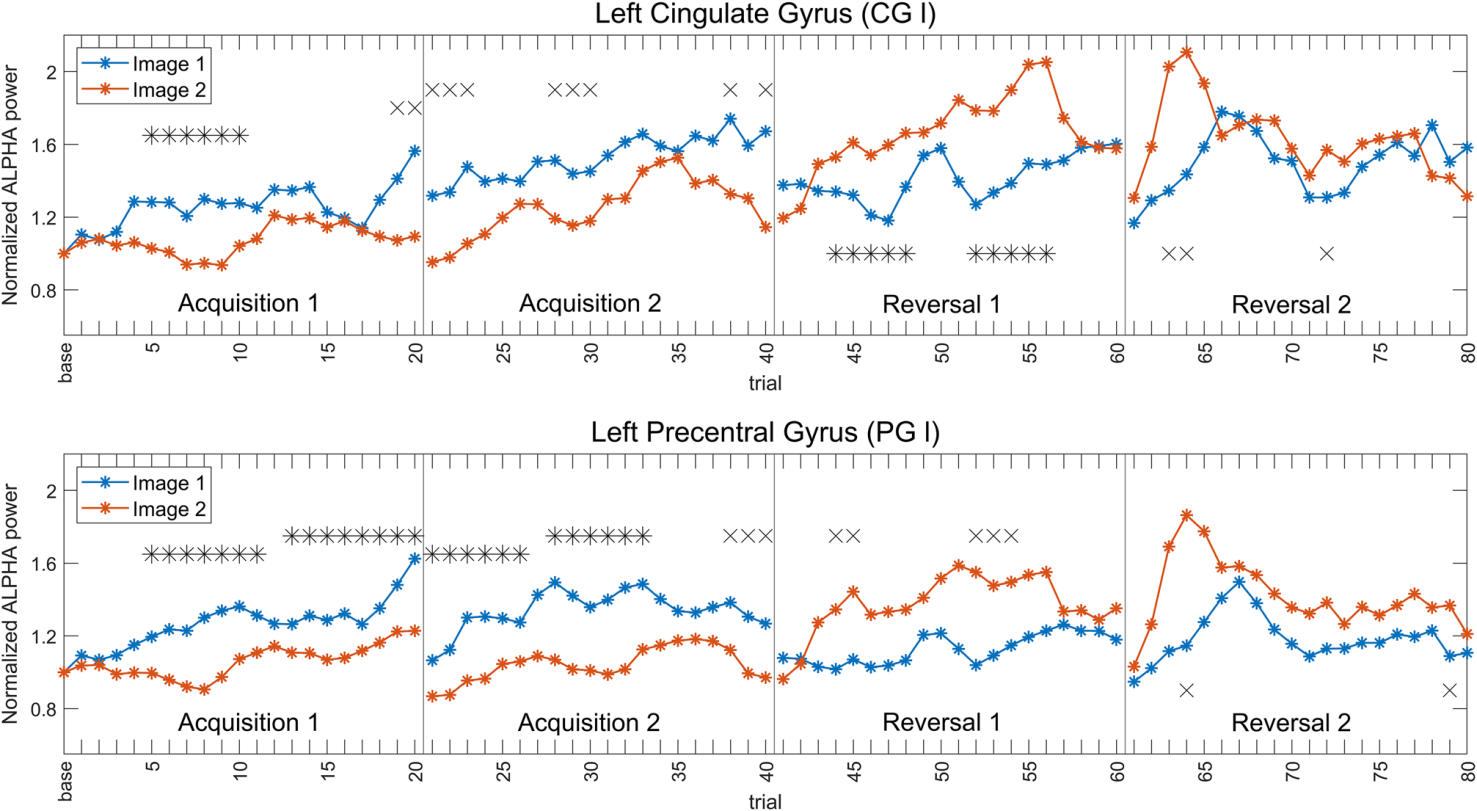
Moving averages (w = 3 trials) of normalized alpha power, trial by trial, for the four blocks. The top row shows the power of the left cingulate gyrus (CG l), the bottom row the power of the left precentral gyrus (PG l). The two images are shown in the same color (blue for Image 1, red for Image 2). Asterisks indicate the presence of corrected statistical significance (*p* < 0.05, cluster-based correction) for that particular trial, while crosses indicate the presence of uncorrected statistical significance (*p* < 0.05). Vertical lines are used to delineate the four different blocks. It is evident that alpha power is always greater during CS- (Image 1 in Acq1 and Acq2; Image 2 in Rev1 and Rev2), and that inversion occurs within three-four reversal trials of the moving average

Figure 8 shows the alpha power moving average for two regions, CG l and PG l. Among all, these two regions have been selected since exhibit the greatest number of significant trials (cluster correction) in the moving average signal. In fact, in almost all trials, alpha power is higher in the CS- (i.e., during the presentation of Image 1 in Acq1 and Acq2, Image 2 in Rev1 and Rev2) than in the CS + , with a clear inversion occurring in the reversal phase. This difference emerges after 3–4 steps of the moving average signal (as evident during Acq1 and Rev1). However, in Rev2, the power difference between the two images is reduced compared with the previous blocks. Indeed, fewer trials exhibit statistically significant differences in this last block. Finally, an abrupt fall in alpha-power is always evident from one block to the next, probably reflecting a more stressful or attentive condition at the beginning of each new block. A similar trend, not shown for brevity and reported in the supplementary material (SI_6), is evident also for the other nine selected regions.

#### Gamma power

Although some significant differences were found in gamma power between Image 1 and Image 2 in some regions and some blocks (more specifically, in Acq1: left anterior cingulate, left cuneus, left orbital gyrus, left subcallosal gyrus; in Acq2: right paracentral lobule, right precentral gyrus, left angular gyrus, left medial frontal gyrus, left superior occipital gyrus, medial precuneus; in Rev1: right inferior occipital gyrus, left cingulate gyrus, medial cingulate gyrus; in Rev2: medial cuneus), these differences never survived the statistical correction (step 1 in Methods section). For this reason, we did not further analyze gamma power changes or perform connectivity analysis in the gamma band.

### Functional connectivity analysis

The previous analysis revealed the presence of several regions that exhibit significant differences between Image 1 and Image 2 power. In this section we investigate, through Granger connectivity, how information is transferred from these regions toward other regions in the brain (i.e., how this increased or decreased power is transferred). Connectivity in the theta band is illustrated considering the connections that emerge from the two regions, CG l and CG m, since both display significant connectivity differences between Image 1 and Image 2. Regarding the alpha rhythm, only connectivity from two areas (CG l and PG l) is shown. In fact, connectivity was evaluated also from the remaining areas but statistical differences between Image 1 and Image 2 were unclear and did not provide any evident network topology.

**Fig. 9:**
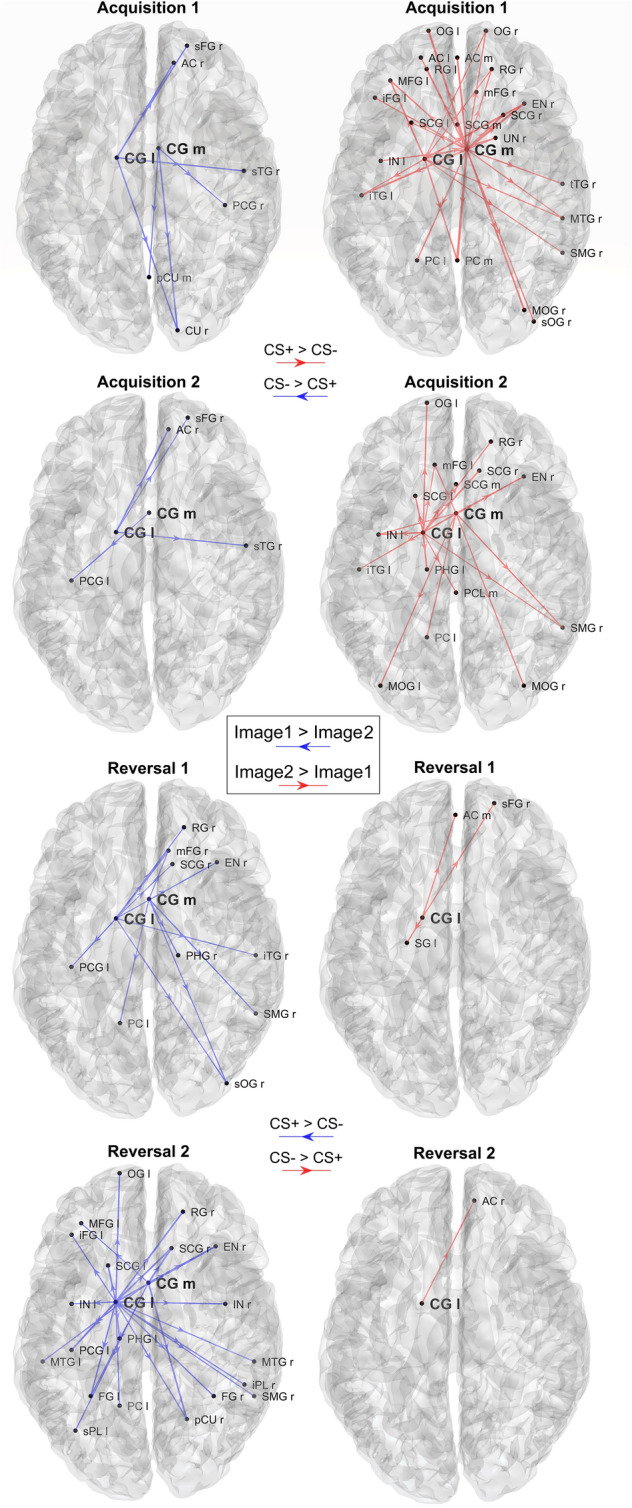
Plots of the strongest differences in connectivity between Image 1 and Image 2, calculated in theta band, block by block. Only connection differences with absolute value greater than 1/4 of the mean are displayed, exiting from the two selected regions CG m and CG l. The left column shows the connections that are greater during the processing of the Image 1 (CS- in acquisition, CS + in reversal; blue directional arrows), whereas the right column shows connections that are greater during processing of Image 2 (CS + in acquisition, CS- in reversal; red directional arrows). In all blocks the connectivity is stronger during CS + [i.e., during the presentation of Image 2 in Acq1 and Acq2 (right column), and Image 1 in Rev1 and Rev2 (left column)], with a clear inversion occurring from acquisition to reversal

**Fig. 10:**
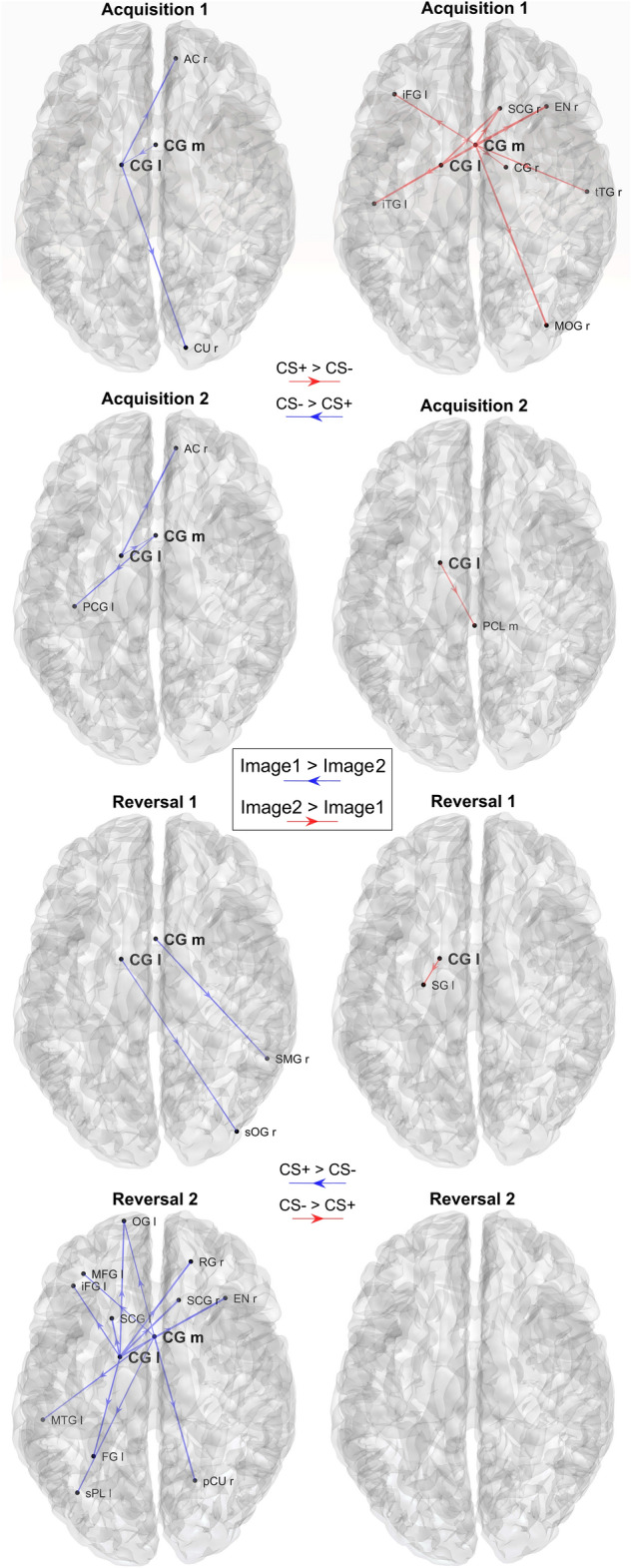
Plots of the significant differences in connectivity between Image 1 and Image 2, calculated in the theta band, block by block. Only the connections exiting from the two selected regions CG m and CG l and which are significantly different between Image 1 and Image 2 in each block (*p* < 0.05, uncorrected) are displayed (sparse matrices). The left column shows the connections that are greater during processing of the Image 1 (CS- in acquisition, CS + in reversal; blue directional arrows), whereas the right column shows connections that are greater during processing of Image 2 (CS + in acquisition, CS- in reversal; red directional arrows). It is worth noting that differences are evident during acquisition 1 and reversal 2 only, with a clear inversion of the connectivity and a strong impact especially in reversal 2

**Fig. 11:**
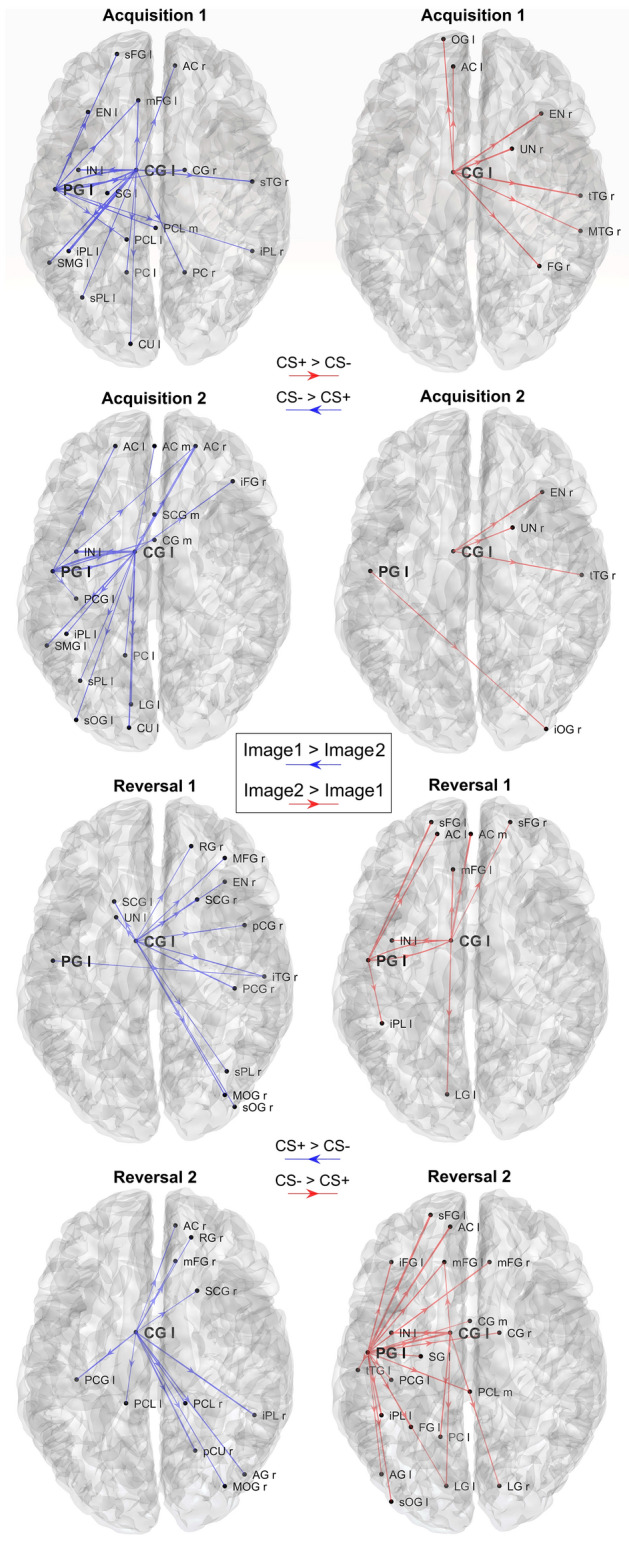
Plots of the strongest differences in connectivity between Image 1 and Image 2, calculated in alpha band, block by block. Only the connection differences with absolute value greater than 1/6 of the mean are displayed, coming from the two selected regions PG l and CG l. The left column shows the connections that are greater during processing of the Image 1 (CS- in acquisition, CS + in reversal; blue directional arrows), whereas the right column shows connections that are greater during processing of Image 2 (CS + in acquisition, CS- in reversal; red directional arrows). In all blocks the connectivity is stronger during CS- [i.e., during the presentation of Image 1 in Acq1 and Acq2 (left column), and Image 2 in Rev1 and Rev2 (right column)], with a clear inversion occurring from acquisition to reversal

**Fig. 12:**
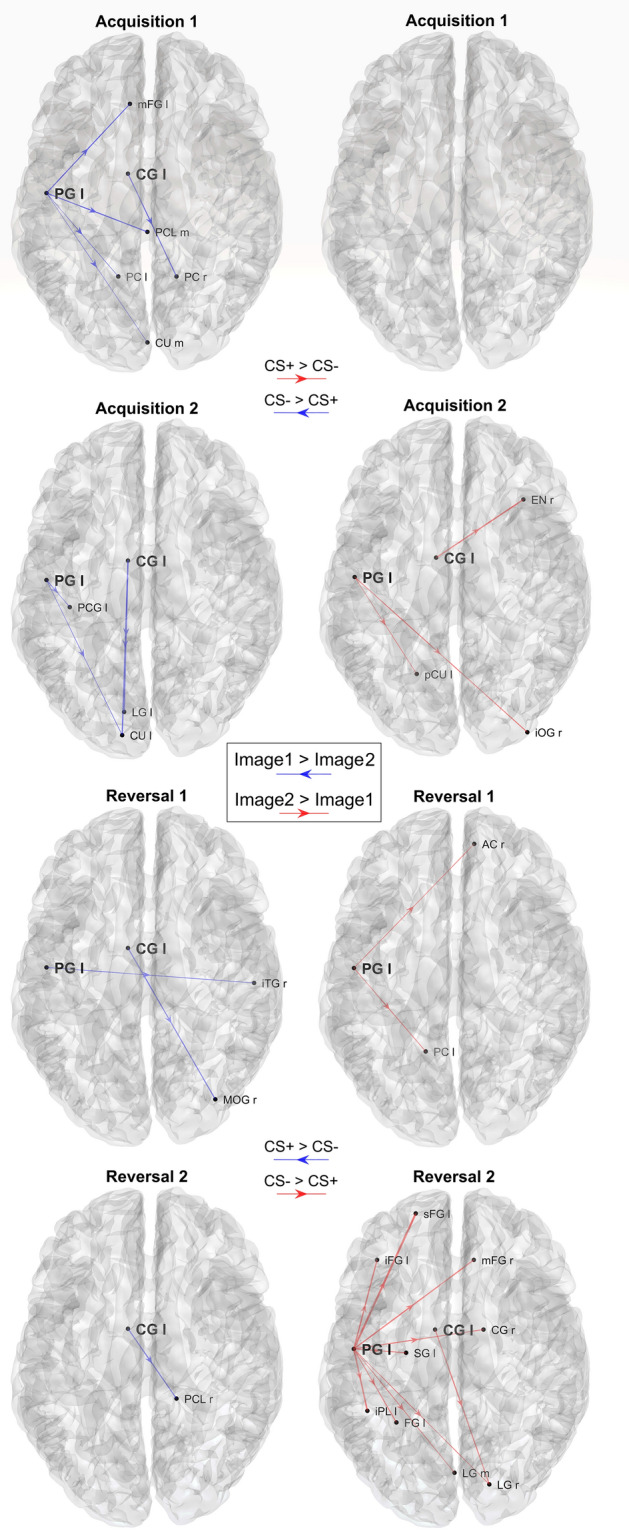
Plots of the significant differences in connectivity between Image 1 and Image 2, calculated in alpha band, block by block. Only the connections coming from the two selected regions PG l and CG l and which are significantly different between Image 1 and Image 2 in each block (*p* < 0.05, uncorrected) are displayed (sparse matrices). The left column shows the connections that are greater during processing of the Image 1 (CS- in acquisition, CS + in reversal; blue directional arrows), whereas the right column shows connections that are greater during processing of Image 2 (CS + in acquisition, CS- in reversal; red directional arrows). It is worth noting that differences in connectivity are evident only concerning PG l during acquisition 1 and reversal 2, with a clear inversion of the connectivity and a strong impact especially in reversal 2

In each of the following Figs. 9, 10, 11, and 12, the first column displays the connections that are stronger during the processing of Image 1 (Image1 > Image2: blue arrows), whereas the second column shows the connections that are stronger during the processing of Image 2 (Image2 > Image1: red arrows). In the first set of plots, we show the connections, which exhibit the largest differences (in absolute value) between Image 1 and Image 2 in each block, independently of the statistical difference (Figs. 9, 11), to show a general trend. Since all connectivity matrices are normalized to 100, and we have a total of 76 × 75 connections, the mean value of the connections is 0.0175. We chose to plot all connection differences that overcome 1/4 of the mean value for theta and 1/6 for alpha band. These different thresholds were chosen because the differences turned out higher in the theta than in the alpha range.

In the second set of plots, only connections that exhibit a significant statistical difference between Image 1 and Image 2 (*p* < 0.05, uncorrected) are displayed (Figs. 10, 12).

#### Theta connectivity

Figure 9 shows the strongest differences in absolute value (greater than 0.0043 = 1/4 of the mean) concerning the connections that exit from the two areas CG l and CG m. In both ROIs and all blocks, the connectivity is stronger during CS + (i.e., during the presentation of Image 2 in Acq1 and Acq2, Image 1 in Rev1 and Rev2), thus mimicking changes in power. An inversion in outgoing connectivity is evident from acquisition to reversal in both regions. Moreover, this difference is more pronounced in the second reversal block than in the first (at odd with the pattern of theta power, whose difference between Images was more evident in reversal 1 than in reversal 2).

In Fig. 10, theta-band connectivity is displayed showing only those connections from the two selected ROIs, which are significantly different between Image 1 and Image 2 (sparse connectivity matrices). As in the non-sparse representation (Fig. 9), a higher number of connections from CG l and CG m can be observed during CS + (i.e., during the presentation of Image 2 in Acq1 and Acq2, Image 1 in Rev1 and Rev2) than CS-, but this difference is evident only in the first acquisition block and in the second reversal block. Finally, the effect in reversal 2 (Image 1 (new CS +) > Image 2 (new CS-), bottom row) is even more pronounced than in acquisition 1.

#### Alpha connectivity

As evident in Fig. 11 (which shows the connections with an absolute value higher than 0.0029 = 1/6 of the mean, emerging from the two selected ROIs), both in the acquisition and reversal, connections arising from the PG l are greater during the CS- stimulus (Image 1 during Acq1 and Acq2, and Image 2 during Rev1 and Rev2), thus mimicking the same behavior as the alpha power. Conversely, the CG l shows a more complex behavior, with some connections being higher during Image 1 and other during Image 2. All figures show an explicit inversion of connections in the acquisition-reversal transition.

In Fig. 12, the same connectivity is displayed, but showing only significantly different connections between Image 1 and Image 2 (i.e., using sparse connectivity matrices). In this graph, a clear difference in connections is still evident in PG l, with connections stronger in CS- (i.e., during the presentation of Image 1 in Acq1 and Acq2, Image 2 in Rev1 and Rev2) than in CS + . However, this effect is mainly limited to the first block (acquisition 1) and becomes even more evident in the final block (reversal 2). Conversely, the differences in connections emerging from CG l are scarcely noticeable when the sparse matrix is used.

### Summary of key findings

Here, a summary of the main results is provided. The scalp-level analysis (Fig. 2) shows a greater theta power during CS + than during CS-, especially in fronto-medial channels, and an increased alpha power during CS- on a broader area, especially involving the left-parietal electrodes. These results are stressed by the study performed at the cortical source level, showing a statistically significant (corrected) increase in theta power during CS + in the left and medial cingulate gyrus (Figs. 3 and 4) and a significant (corrected) increase in alpha power during CS- in the left motor and somatosensory areas (contralateral to the shock, Figs. 6 and 7). It is worth noting that these changes remain consistent during reversal (considering the new CS + and the new CS-), although significant only in the first block. Furthermore, concerning the theta response, reversal occurs just after one trial (Fig. 5), whereas three-four trials are necessary to observe a reversal in alpha power (Fig. 8).

Connectivity analysis, limited to the outgoing connections from the most significant regions revealed by the previous study, shows stronger theta connectivity emerging from the left cingulate gyrus during CS + (Figs. 9 and 10) and stronger connectivity arising from the left precentral gyrus during CS- (Figs. 11 and 12). Both are also evident during reversal. Interestingly, these connectivity differences between CS + and CS- are higher during the last reversal block, when differences in power density become elusive, suggesting a role for synaptic plasticity in reversal.

## Discussion

The objective of this work was to investigate the mechanisms of fear acquisition and reversal in healthy human volunteers, laying particular emphasis on the contribution of brain rhythms. To this end, we used high-density scalp EEG and SCR measurements during acquisition and reversal of Pavlovian fear conditioning. Even though fear conditioning has been the subject of many studies in recent years, our work introduces some aspects of novelty: first, we compared the pattern of brain rhythms during acquisition and reversal in all cortical ROIs, to point out similarities and differences between the two phases; second, we looked at the effect of time on fear learning, to point out in which phases rhythms play a pivotal role, and in which phases their role is less evident; third, we analyzed changes in output connectivity from the regions of interest and in the frequency bands more robustly implicated in the fear learning response. Our results confirm several aspects of the literature and introduce new elements that can help clarify the involvement of brain rhythms in Pavlovian fear conditioning.

### Theta rhythm

A first significant result concerns the role of the theta rhythm in fear acquisition. Our data show an increase in theta power in the left-mid cingulate cortex in response to the CS + stimuli. This role is particularly marked during the first block of acquisition (see also Table 2) but remains evident (although less pronounced) during the second acquisition block and the first reversal block. Noticeably, in the second reversal block, although CS + theta power is still higher than the CS- theta power, the difference becomes less substantial, and the role of theta rhythm progressively attenuates. The same pattern is confirmed by the trial-by-trial analysis using the moving average signal, which provides additional attractive cues. Indeed, at the beginning of the first acquisition block, theta power in the cingulate cortex increases abruptly both during CS + and CS-, probably signaling an alert phase of the experiment. Then, after just 1–2 trials, theta power differentiated between CS + and CS-. It is worth noting that this difference becomes maximally evident during the mid-period of the acquisition phase and then progressively declines toward the end of acquisition 2. The rapidity of the theta response is confirmed by looking at the reversal phase: just 1–2 shock-associated trials are sufficient to significantly increase theta power in response to the new aversive image.

These patterns, taken together, suggest that theta oscillations in the cingulate cortex signal the presence of a new aversive event, and this pattern is already evident during the first trials of the learning phase. However, these results also underline some differences between acquisition and reversal. Although the theta power difference between CS + and CS- develops promptly, it remains weaker during the reversal phase than in the previous acquisition phase, especially during the second reversal period. Overall, it seems that the theta rhythm signals the novelty of the aversive event and then declines when the association has been established, with this decline especially evident in the second reversal period, making power difference during reversal less manifest than during acquisition. This result agrees with a recent finding by (Taub et al. 2018): the authors suggest that the increase in theta power during aversive conditioning is correlated with the magnitude of conditioned responses but declines once the association is stabilized. Similarly, (Ridderbusch et al. 2021) observed a temporary increase in neural activation in the anterior cingulate cortex after re-exposure to the US after extinction training and suggested that this is associated with exploratory behavior, signaling changes in US-expectancy and arousal ratings.

Several studies underline the implication of the anterior (mid or dorsal) cingulate cortex in fear acquisition (Milad et al. 2007a; Toyoda et al. 2011; Mueller et al. 2014; Feng et al. 2014; Bierwirth et al. 2021). These results substantially agree with ours. Our study adopted the subdivision among ROIs illustrated in Table 1, according to the atlas used by LORETA-KEY©®. Using this atlas, we found significant theta power differences in the cingulate gyrus (left and medial). According to this atlas, the cingulate gyrus includes, among the others, the posterior portions of the Brodmann regions 24 and 32, which are traditionally ascribed to the ACC. However, it is worth noting that the Atlas also includes two other “cingulate” regions named “anterior cingulate” and “posterior cingulate” (see Table 1). In particular, the region called “anterior cingulate” includes the most anterior portions of areas 24 and 32. This subdivision agrees with the functional description of the cingulate gyrus proposed by (Vogt 2009). The author states that “the greatest number of “fear” activations occur in the anterior part of the midcingulate cortex MCC and not in ACC”, The first roughly corresponds to the posterior portions of the Brodmann areas 32 and 24, i.e. to the CG region used in the present atlas.

Many other results in rodents, primates, and humans underline the impact of theta oscillations in fear learning. Synchronization at theta frequencies is suggested to characterize activity in amygdala-hippocampal pathways associated with the consolidation of fear memory (Pape et al. 2005) and to represent a general mechanism of fear learning across species (Chen et al. 2021). A shared hypothesis is that the theta rhythm develops in the amygdala and hippocampus limbic system and is then transmitted to the ventromedial prefrontal cortex and to the anterior midcingulate cortex to synchronize ACC activity, and to transfer error signal information to support memory formation (Verbeke et al. 2021). Indeed, the anterior midcingulate cortex receives afferents from the amygdala (Vogt and Pandya 1987; Vogt 2005). Furthermore, synchronized frontomedial theta oscillations are a potential mechanism to support memory communication between brain regions (Sperl et al. 2019).

As to the last point (i.e., theta transmission and synchronization), a novel significant result in our study concerns the pattern of connections emerging from the previous two regions (CG l and CG m). We observed that Granger connectivity in the theta range is stronger during CS + than during CS-, and these differences are also evident during reversal. A possible interpretation is that the increased theta power in these regions is then transmitted to other areas of the brain, thus producing a generalized theta synchronization, subserving the retrieval of fear responses, or a general process of adaptive control of an unpleasant event (see also Shackman et al. 2011; Mueller et al. 2014)). Interestingly, if attention is focused only on connections statistically different between CS + and CS- (not only to the absolute differences), the increase in connectivity during CS + is especially evident during the first acquisition and second reversal blocks. Hence, a puzzling phenomenon is that theta power differences between CS + and CS- decline in reversal 2, whereas connectivity differences become more evident in the same block. Thus, functional connectivity does not simply reflect a change in power of the theta rhythm transmitted to other regions but may also depend on an effective alteration of synapses, especially in the last portion of the experiment. Further studies are needed to clarify this crucial point.

### Alpha rhythm

A significant observation emerging from our data is that alpha power changes are less localized than the changes in theta power and involve a more extensive network mainly located in portions of the posterior frontal cortex and parietal lobes, with a predominance in the left hemisphere. Some of these zones (the precentral gyrus, paracentral lobule and inferior parietal lobule) are implicated in motor and sensory innervation. As expected, alpha power is smaller during CS + than CS- in all these regions, reflecting a condition of greater arousal. However, it is worth noting that alpha power is higher than baseline during all phases of the experiment, probably since the initial period of the experiment (before any trial) was felt as the most stressful condition for the participants, possibly due to uncertainty of what will happen next. Alpha power differences are more evident in the acquisition phase than in the reversal phase, and, in the cingulate gyrus, alpha power exhibits a progressive increase during the experiment, suggesting increasing relaxation of the participants over the course of the experiment.

The pattern of alpha power changes during fear conditioning was investigated in detail by (Chien et al. 2017). The authors observed a significant alpha event-related desynchronization (ERD; i.e., a decrease in power) at parietal and occipital channels, hence over sensory structures related to (visual) CS processing. These changes were especially evident in the early phase of the stimulus train, reflecting a difference between the early and late stages of acquisition. By comparing their results with SCR data, the authors concluded that alpha power changes mainly reflect the valence and salience of the stimulus, i.e., the ability of CS to capture attention and motivate behavior. However, at odds with our results, the authors did not find significant differences in alpha power between CS + and CS-. Differences between our results and those by Chien et al. can be explained by thinking that these authors mainly focused on the magnitude of alpha ERD, which is maximal in the occipital regions, implicated in the visual processing of the external stimuli. Conversely, we focused on statistical differences between CS + and CS-, concentrated in parietal and posterior frontal regions, i.e., in the zones mainly involved in tactile and motor processing.

A significantly greater alpha-power suppression for threat-conditioned CS + than CS- was observed by (Panitz et al. 2019) at the scalp sites corresponding to visuocortical (i.e., occipital) areas. These differences likely indicate a sustained allocation of visual attention to the conditioned threat cue. Our results replicate and extend the results by (Panitz et al. 2019), demonstrating a similar effect at the cortical source level and on a broader set of regions (not only visuocortical but also somatosensory and motor).

Since the precentral gyrus is primarily involved in motor processing, a decreased alpha during CS + in this zone may reflect greater motor activation in preparation for an escape (for instance, preparation of movement of the right arm where the shock is delivered). Indeed, alpha ERD reflects the gradual release of inhibition associated with the emergence of a task-response. In contrast, an increase in alpha oscillations (event related synchronization, ERS) is observed with the CS-. Alpha ERS is commonly ascribed to idling or suppressing activity in task-irrelevant sites (Klimesch et al. 2007). Hence, our result supports the idea that alpha power changes observed in parietal and posterior frontal zones primarily reflect a preparative response to an action (during CS +) or a partial idling (during CS-) of the same activity.

Finally, it is worth noting that the time response of this alpha pattern is slower than that of the theta response: as evident looking at the moving average, alpha ERS during CS- requires 3–4 trials to develop. Another interesting aspect is that alpha power exhibits a drastic fall (ERD) at the beginning of any new block, reflecting greater attention/arousal due to the unfamiliar new conditions. Then alpha power progressively increases (especially in CS-), reducing the response in motor areas.

The connectivity pattern in the alpha band further underlines the pivotal role played by the left precentral gyrus. Stronger outflow connectivity is evident in this area during CS- than during CS + , reflecting the higher alpha power transmitted towards other occipital, parietal and frontal regions. It is worth noting that the reverse of this connectivity pattern is relatively slow, being maximally evident during reversal 2 than during reversal 1. This phenomenon is similar to what has already been observed for the theta connectivity from the left and medial cingulate cortex. In other terms, connectivity changes mature more slowly during reversal than during acquisition, becoming fully evident in the second reversal block. This pattern probably reflects synaptic changes necessary to overcome a previous pattern of connectivity developed during the acquisition phase. Indeed, as shown in recent modeling studies (Ursino et al. 2020; Ricci et al. 2021) functional connectivity mainly reflects the amount of information transmitted from one region to another: the latter can depend both on the power in the source region and on the strength of the effective connectivity linking the two regions.

Unexpectedly, the pattern of alpha-band connectivity emerging from the left cingulate cortex apparently contradicts the pattern of alpha power: in fact, many of these connections are higher during CS + than during CS-, i.e., in conditions of ERD. We do not have a definitive explanation for this pattern. However, we suspect that these seemingly anomalous connectivity patterns reflect non-linear phenomena and are strongly affected by changes in theta power (which, as demonstrated above, are significant in the left cingulate cortex and are higher in CS + than CS-). In previous papers (Ursino et al. 2020, 2021; Ricci et al. 2021) using a neural mass model as ground truth and comparing the actual connectivity values in the model with those obtained with methods for functional connectivity assessment, we demonstrated that non-linear phenomena play a significant role in connectivity estimation, resulting in possible interference between frequency bands and alterations in the connectivity values.

### Gamma rhythm

There is a consensus in the literature that gamma power is implicated in inhibiting a previously acquired fear response (Stujenske et al. 2014; Courtin et al. 2014; Fenton et al. 2016). (Mueller et al. 2014) observed that, in humans, vmPFC gamma activity differs between extinguished CS + and CS-. The role of the vmPFC in extinction is further supported by neuroimaging studies (Milad et al. 2007b). However, (Schiller et al. 2008) pointed out that reversal is a more complex process than extinction. Using fMRI, these authors observed that, during reversal, the activity in the vmPFC signals the presence of a safe stimulus (hence the new CS- in reversal, previously CS + during acquisition), which can be interpreted as an unexpected reward.

According to the studies mentioned above, a significant gamma activity in the vmPFC was expected in reversal; however, in our research, we were unable to find any corrected statistical difference in gamma between CS + and CS- during any phase of the experiment. For this reason, gamma activity was not further analyzed.

## Conclusions

The results obtained in this study confirm several observations of previous studies and add new aspects. (i) Increase in theta rhythm power occurs in the mid portion of the cingulate cortex during CS + and is associated with an increase in outflow connectivity. This may reflect a rhythm from the amygdala and hippocampus, which is then transmitted to other cortical regions allowing a fast theta synchronization, as supported by our Granger Causality analysis. Theta synchronization may play a pivotal role during the acquisition of fear conditioning. (ii) Alpha power ERD during CS + and alpha power ERS during CS- occur mainly in the left posterior frontal and parietal cortex, with the most substantial evidence in the left precentral gyrus. These two phenomena may reflect an excitation of these motor areas (movement preparation) in case of an aversive stimulus and a progressive inhibition of these areas in case of a safe stimulus, respectively. (iii) The dynamics of theta power changes appear faster than those of the alpha rhythm, reflecting a trial-by-trial basis. (iv) All the previous phenomena are present during acquisition and reversal, but differences between CS + and CS- are less prominent in the reversal phases. This may be due to the difficulty of overcoming a previously acquired memory. (v) Changes in power are associated with increased Granger connectivity emerging from the areas involved. Unexpectedly, these connectivity changes are also strongly evident in the second reversal block when power differences are attenuated. This phenomenon may reflect changes in real connectivity instead of simple changes in oscillation power and requires further study.

## Supplementary Information

Below is the link to the electronic supplementary material.

Supplementary file1 (DOCX 2171 KB)

Supplementary file2 (XLS 585 KB)

Supplementary file3 (DOCX 251 KB)

Supplementary file4 (DOCX 10770 KB)

Supplementary file5 (DOCX 971 KB)

Supplementary file6 (DOCX 4504 KB)

## Data Availability

The datasets generated and analyzed during the current study are available from the corresponding author undue reservation.
