# Supplementary material for: Changes in brain rhythms and connectivity tracking fear acquisition and reversal
Source: Brain Struct Funct. 2023 May 2;228(5):1259–81. doi: 10.1007/s00429-023-02646-7 (PMC10250514; doi:10.1007/s00429-023-02646-7)
Supplement: Supplementary file 1 — Supplementary file1 (DOCX 2171 KB) [file 429_2023_2646_MOESM1_ESM.docx]

**SUPPLEMENTARY INFORMATION 1**

**CHANGES IN BRAIN RHYTHMS AND CONNECTIVITY TRACKING FEAR ACQUISITION AND REVERSAL**

Gabriele Pirazzini^1^*, Francesca Starita^2^, Giulia Ricci^1^, Sara Garofalo^2^, Giuseppe di Pellegrino^2^, Elisa Magosso^1^, Mauro Ursino^1^

1 Department of Electrical, Electronic, and Information Engineering "Guglielmo Marconi", University of Bologna, 47521 Cesena, Italy

2 Center for Studies and Research in Cognitive Neuroscience, Department of Psychology, University of Bologna, 40126 Bologna, Italy

* Corresponding author – Gabriele Pirazzini: [gabriele.pirazzini3@unibo.it](mailto:gabriele.pirazzini3@unibo.it)

Address: Department of Electrical, Electronic, and Information Engineering "Guglielmo Marconi", Area di Campus Cesena, Via Dell'Università 50, I 47521 Cesena FC

This section of the supplementary information shows a flow-chart representing the main processing steps of the raw EEG signal (but see also *‘EEG recording and processing’*, in the *Methods* section, for more details).

**

**

***(SI_1)* Fig.1** Main processing steps carried out on the raw EEG data and main analyses carried out thereafter. The raw signal is first down-sampled (from 1 kHz to 500 Hz) and then filtered through a 1-60 Hz bandpass filter and a 50 Hz notch filter. Then, 160 epochs of 6s duration (from onset stimulus to eventual shock administration) are extracted (40 trials for each block) and eventually bad channels are identified. All these steps are carried out through custom-made scripts in MATLAB software. The EEGLAB toolbox is then used to perform Independent Component Analysis (ICA) in order to identify and remove artifacts. Through the LORETA software (and eLORETA method) we carried out cortical source estimation, subdividing the cortex into 6239 voxels. Then, these 6239 voxels are merged into 76 Regions of Interest (according to LORETA's atlas, see also Table 1 in the main text and supplementary information SI_2). At this stage, two main analyses are performed on the signal: i) power computation in the theta, alpha and gamma bands; ii) functional connectivity analysis via Granger Causality estimator.
