# Supplementary material for: Changes in brain rhythms and connectivity tracking fear acquisition and reversal
Source: Brain Struct Funct. 2023 May 2;228(5):1259–81. doi: 10.1007/s00429-023-02646-7 (PMC10250514; doi:10.1007/s00429-023-02646-7)
Supplement: Supplementary file 3 — Supplementary file3 (DOCX 251 KB) [file 429_2023_2646_MOESM3_ESM.docx]

**SUPPLEMENTARY INFORMATION 3**

**CHANGES IN BRAIN RHYTHMS AND CONNECTIVITY TRACKING FEAR ACQUISITION AND REVERSAL**

Gabriele Pirazzini^1^*, Francesca Starita^2^, Giulia Ricci^1^, Sara Garofalo^2^, Giuseppe di Pellegrino^2^, Elisa Magosso^1^, Mauro Ursino^1^

1 Department of Electrical, Electronic, and Information Engineering "Guglielmo Marconi", University of Bologna, 47521 Cesena, Italy

2 Center for Studies and Research in Cognitive Neuroscience, Department of Psychology, University of Bologna, 40126 Bologna, Italy

* Corresponding author – Gabriele Pirazzini: [gabriele.pirazzini3@unibo.it](mailto:gabriele.pirazzini3@unibo.it)

Address: Department of Electrical, Electronic, and Information Engineering "Guglielmo Marconi", Area di Campus Cesena, Via Dell'Università 50, I 47521 Cesena FC

This section of the supplementary information reports a figure showing the SCR findings.


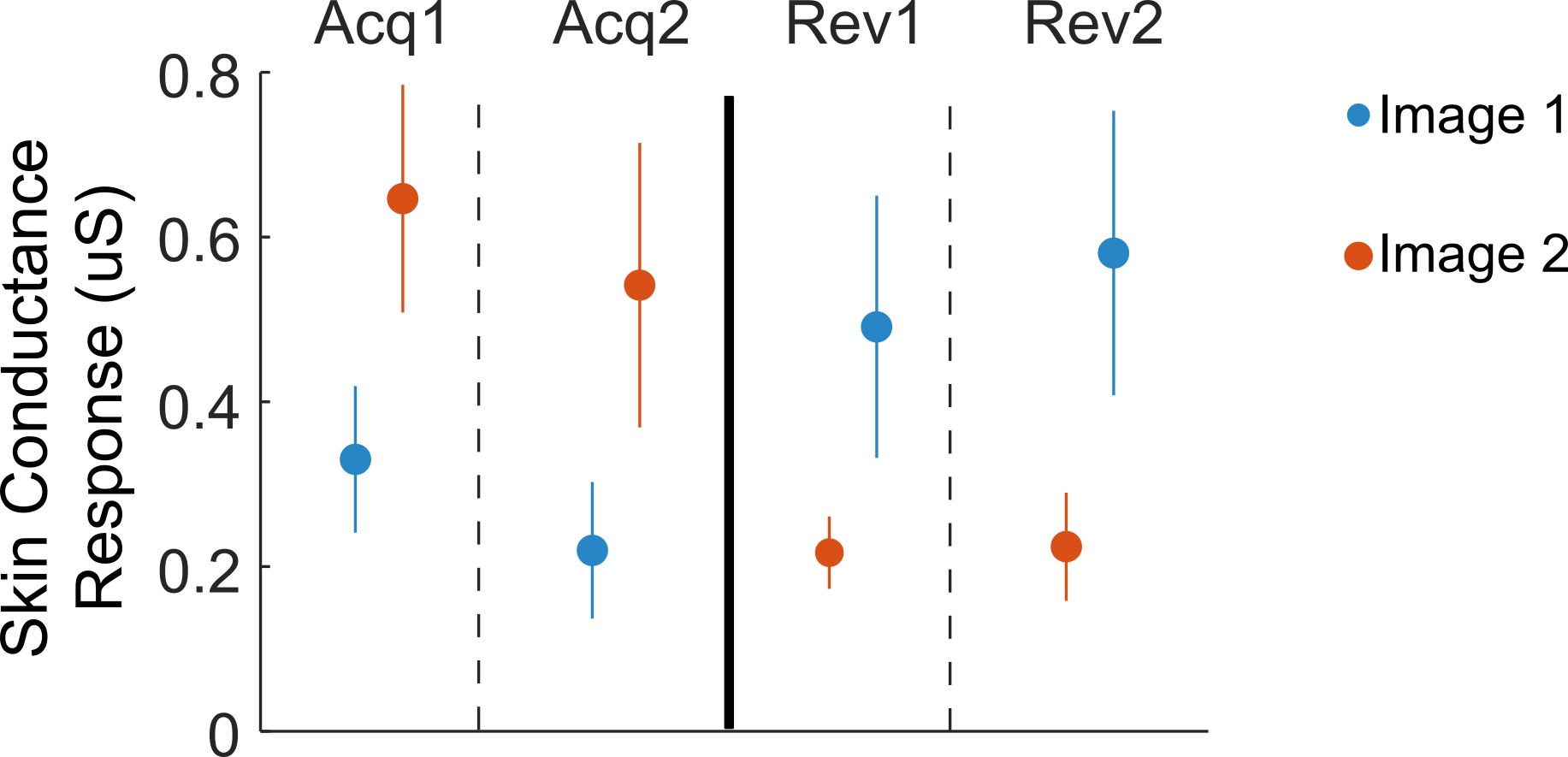


***(SI_3)* Fig 1** The plot shows group means (circles), and 95% confidence intervals (vertical lines) of square rooted skin conductance response to Image 1 and Image 2, as a function of the experimental phase (acquisition 1 & 2, or reversal 1 & 2). 95%CI were corrected for within-subjects designs
