# Supplementary material for: Changes in brain rhythms and connectivity tracking fear acquisition and reversal
Source: Brain Struct Funct. 2023 May 2;228(5):1259–81. doi: 10.1007/s00429-023-02646-7 (PMC10250514; doi:10.1007/s00429-023-02646-7)
Supplement: Supplementary file 4 — Supplementary file4 (DOCX 10770 KB) [file 429_2023_2646_MOESM4_ESM.docx]

**SUPPLEMENTARY INFORMATION 4**

**CHANGES IN BRAIN RHYTHMS AND CONNECTIVITY TRACKING FEAR ACQUISITION AND REVERSAL**

Gabriele Pirazzini^1^*, Francesca Starita^2^, Giulia Ricci^1^, Sara Garofalo^2^, Giuseppe di Pellegrino^2^, Elisa Magosso^1^, Mauro Ursino^1^

1 Department of Electrical, Electronic, and Information Engineering "Guglielmo Marconi", University of Bologna, 47521 Cesena, Italy

2 Center for Studies and Research in Cognitive Neuroscience, Department of Psychology, University of Bologna, 40126 Bologna, Italy

* Corresponding author – Gabriele Pirazzini: [gabriele.pirazzini3@unibo.it](mailto:gabriele.pirazzini3@unibo.it)

Address: Department of Electrical, Electronic, and Information Engineering "Guglielmo Marconi", Area di Campus Cesena, Via Dell'Università 50, I 47521 Cesena FC

This section of the supplementary information reports two figures, similar to *Fig. 3* and *Fig. 6* of the main text, highlighting only the statistically significant (corrected) regions, in each different block.

Please note how these figures show all regions that survive correction (in the corresponding block), while the regions analysed in detail in the present work are those identified (among those shown here in the figures) following the two-step method in the subsection *‘Cortical power computation’*.


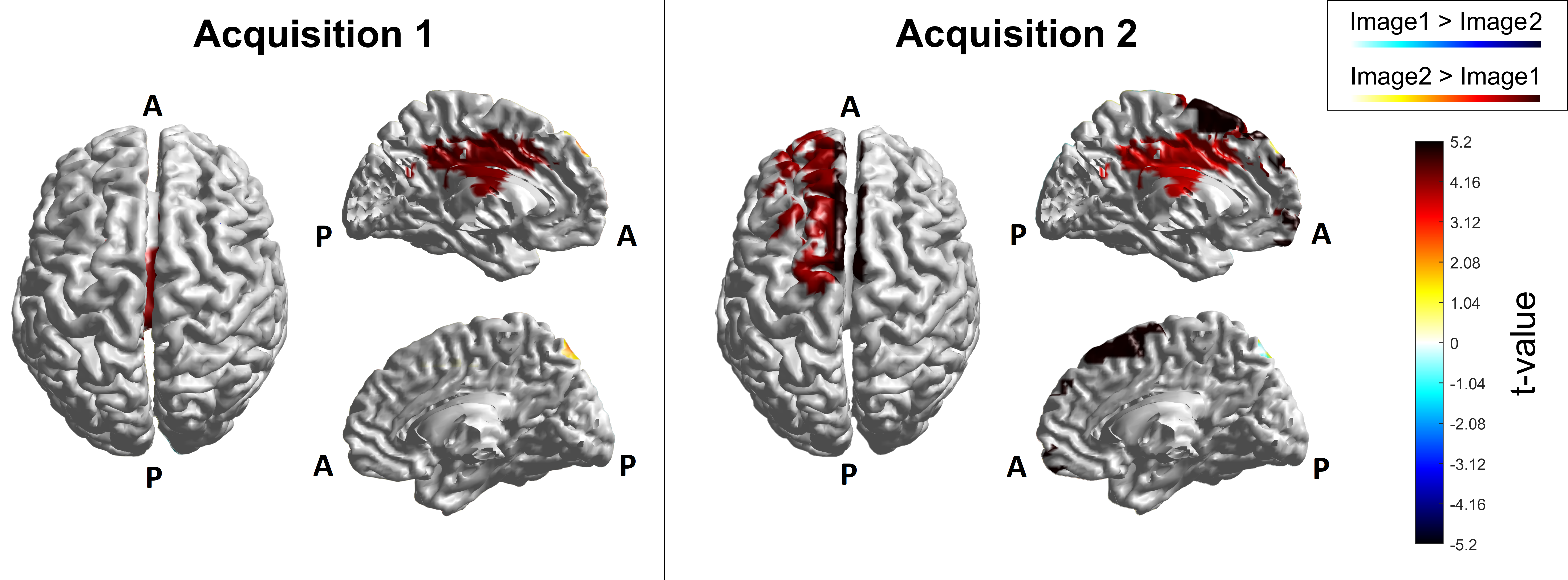


***(SI_4)* Fig. 1** Student's ‘t’ values, resulting from the statistical comparison between the two images, carried out on theta mean power, in the four blocks. Only significant (corrected) ROIs are shown. Please refer to Table 2 in the main text for a list of these regions. In each panel, the left column represents the top view of the cerebral cortex while the right column represents the medial left (top) and the medial right (bottom) view of the cerebral cortex. Letter ‘A’ stands for ‘Anterior’, letter ‘P’ for ‘Posterior’. The color bar corresponds to uncorrected t-values. Positive values (colors tending toward red) indicate higher power during the visualization of Image 2 (CS+ in acquisition, CS- in reversal). No region survives statistical correction in the reversal phase

**
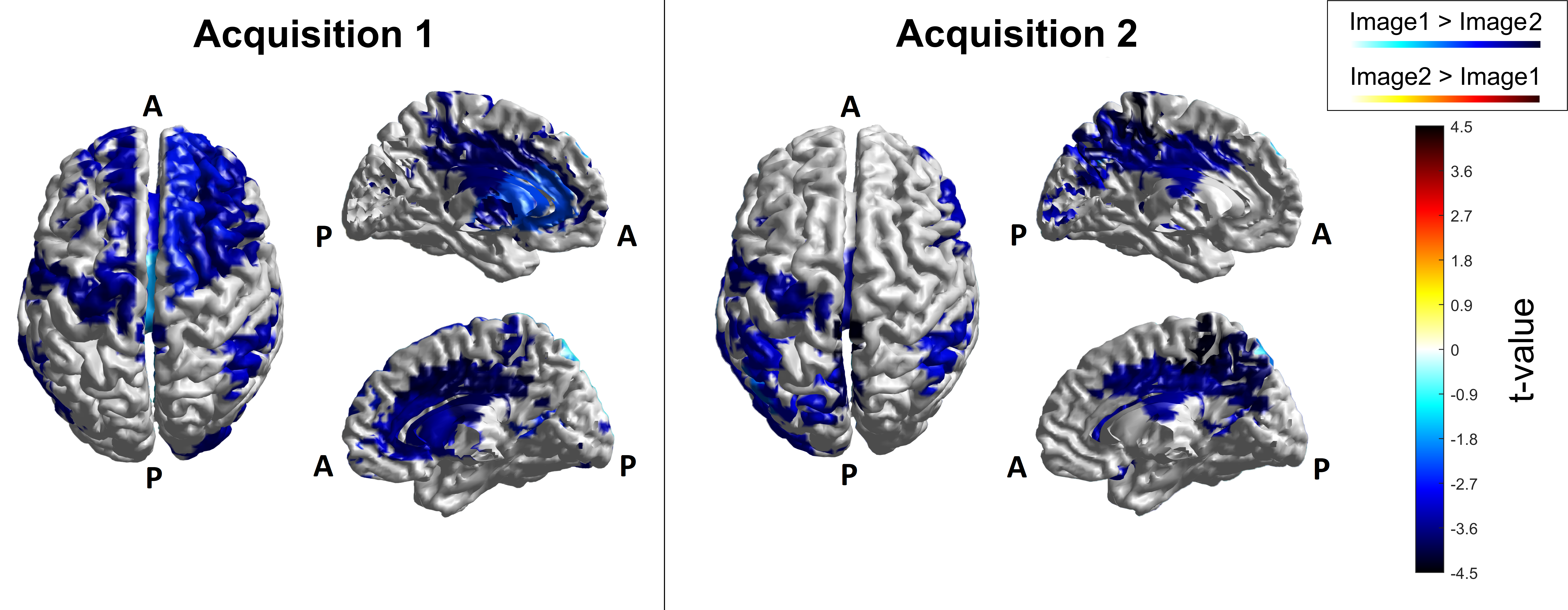
**

***(SI_4)* Fig. 2** Student's ‘t’ values, resulting from the statistical comparison between the two images, carried out on alpha mean power, in the four blocks. Only significant (corrected) ROIs are shown. Please refer to Table 3 in the main text for a list of these regions. In each panel, the left column represents the top view of the cerebral cortex while the right column represents the medial left (top) and the medial right (bottom) view of the cerebral cortex. Letter ‘A’ stands for ‘Anterior’, letter ‘P’ for ‘Posterior’. The color bar corresponds to uncorrected t-values. Negative values (colors tending toward blue) indicate higher power during the visualization of Image 1 (CS- in acquisition, CS+ in reversal). No region survives statistical correction in the reversal phase
