# Supplementary material for: Changes in brain rhythms and connectivity tracking fear acquisition and reversal
Source: Brain Struct Funct. 2023 May 2;228(5):1259–81. doi: 10.1007/s00429-023-02646-7 (PMC10250514; doi:10.1007/s00429-023-02646-7)
Supplement: Supplementary file 5 — Supplementary file5 (DOCX 971 KB) [file 429_2023_2646_MOESM5_ESM.docx]

**SUPPLEMENTARY INFORMATION 5**

**CHANGES IN BRAIN RHYTHMS AND CONNECTIVITY TRACKING FEAR ACQUISITION AND REVERSAL**

Gabriele Pirazzini^1^*, Francesca Starita^2^, Giulia Ricci^1^, Sara Garofalo^2^, Giuseppe di Pellegrino^2^, Elisa Magosso^1^, Mauro Ursino^1^

1 Department of Electrical, Electronic, and Information Engineering "Guglielmo Marconi", University of Bologna, 47521 Cesena, Italy

2 Center for Studies and Research in Cognitive Neuroscience, Department of Psychology, University of Bologna, 40126 Bologna, Italy

* Corresponding author – Gabriele Pirazzini: [gabriele.pirazzini3@unibo.it](mailto:gabriele.pirazzini3@unibo.it)

Address: Department of Electrical, Electronic, and Information Engineering "Guglielmo Marconi", Area di Campus Cesena, Via Dell'Università 50, I 47521 Cesena FC

This section of supplementary information shows the normalized mean alpha power graphs for the five regions not shown in the main text (see **Cortical sources power analysis – *Alpha***, in the *Results* section).

**
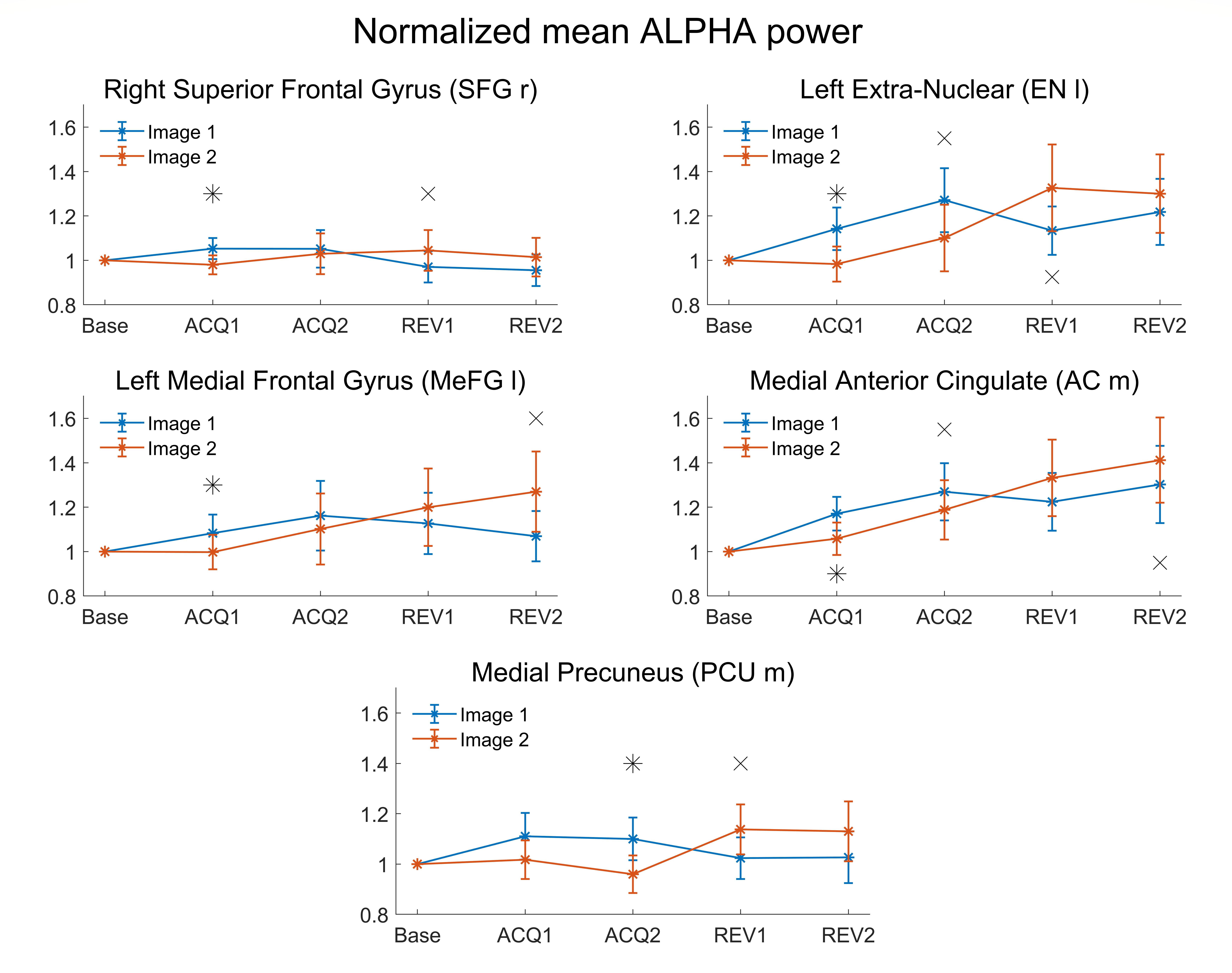
**

***(SI_5)* Fig.1** Normalized mean power in the alpha band, for all four blocks and both images, in the right superior frontal gyrus (SFG r), left extra-nuclear (EN l), left medial frontal gyrus (MeFG l), medial anterior cingulate (AC m) and medial precuneus (PCU m). Results for Image 1 are depicted in blue and those for Image 2 in red, accompanied in each block by the respective SEM bar. Asterisks indicate the presence of corrected statistical significance (p<0.05, false discovery rate correction) in the specific block, while crosses denote the presence of a statistical significance (p<0.05, uncorrected) which does not survive correction for multiple comparisons. It is well evident the power inversion in passing from acquisition to reversal, i.e., alpha power is always greater during CS- (Image 1 in Acq1 and Acq2; Image 2 in Rev1 and Rev2) than CS+
