# Supplementary material for: Changes in brain rhythms and connectivity tracking fear acquisition and reversal
Source: Brain Struct Funct. 2023 May 2;228(5):1259–81. doi: 10.1007/s00429-023-02646-7 (PMC10250514; doi:10.1007/s00429-023-02646-7)
Supplement: Supplementary file 6 — Supplementary file6 (DOCX 4504 KB) [file 429_2023_2646_MOESM6_ESM.docx]

**SUPPLEMENTARY INFORMATION 6**

**CHANGES IN BRAIN RHYTHMS AND CONNECTIVITY TRACKING FEAR ACQUISITION AND REVERSAL**

Gabriele Pirazzini^1^*, Francesca Starita^2^, Giulia Ricci^1^, Sara Garofalo^2^, Giuseppe di Pellegrino^2^, Elisa Magosso^1^, Mauro Ursino^1^

1 Department of Electrical, Electronic, and Information Engineering "Guglielmo Marconi", University of Bologna, 47521 Cesena, Italy

2 Center for Studies and Research in Cognitive Neuroscience, Department of Psychology, University of Bologna, 40126 Bologna, Italy

* Corresponding author – Gabriele Pirazzini: [gabriele.pirazzini3@unibo.it](mailto:gabriele.pirazzini3@unibo.it)

Address: Department of Electrical, Electronic, and Information Engineering "Guglielmo Marconi", Area di Campus Cesena, Via Dell'Università 50, I 47521 Cesena FC

This section of Supplementary Information shows the normalized alpha power moving average graphs for the nine regions not shown in the main text (see **Cortical sources power analysis – *Alpha***, in the *Results* section).

Please note how the following images do not show the results of the statistical analysis (asterisks or crosses at particular trials, see main text). Nevertheless, the statistical analysis was still performed and the total number of significant (corrected/uncorrected) trials/clusters is lower for all of the following areas than for the areas shown in the main text (CG l and PG l).


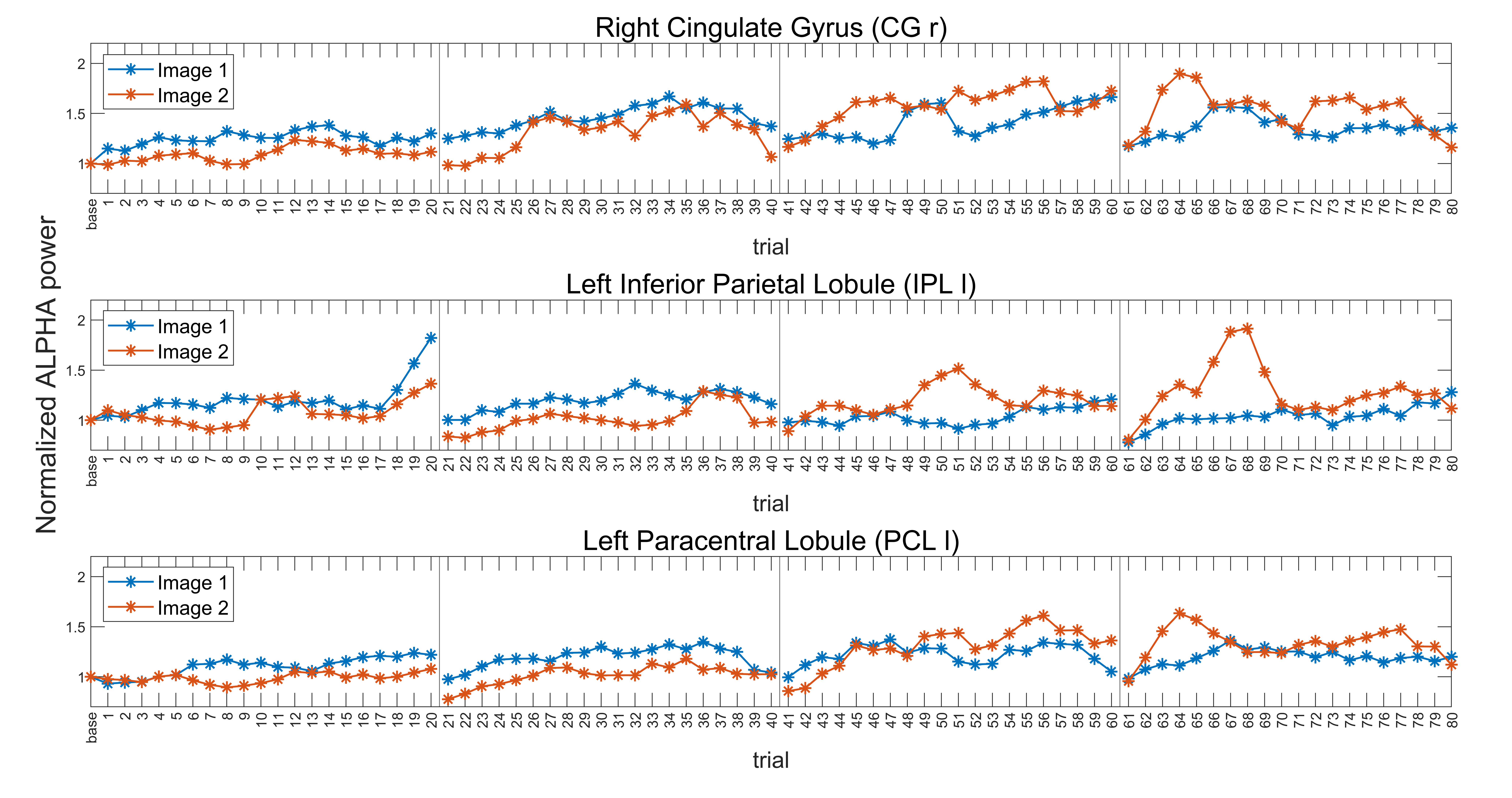


***(SI_6)* Fig. 1** Moving averages (w=3 trials) of normalized alpha power, trial by trial, for the four blocks. The top row shows the normalized power of the right cingulate gyrus (CG r), the central row the normalized power of the left inferior parietal lobule (IPL l), and the bottom row the normalized power of the left paracentral lobule (PCL l). The two images are shown in the same color (blue for Image 1, red for Image 2). Vertical lines are used to delineate the four different blocks.


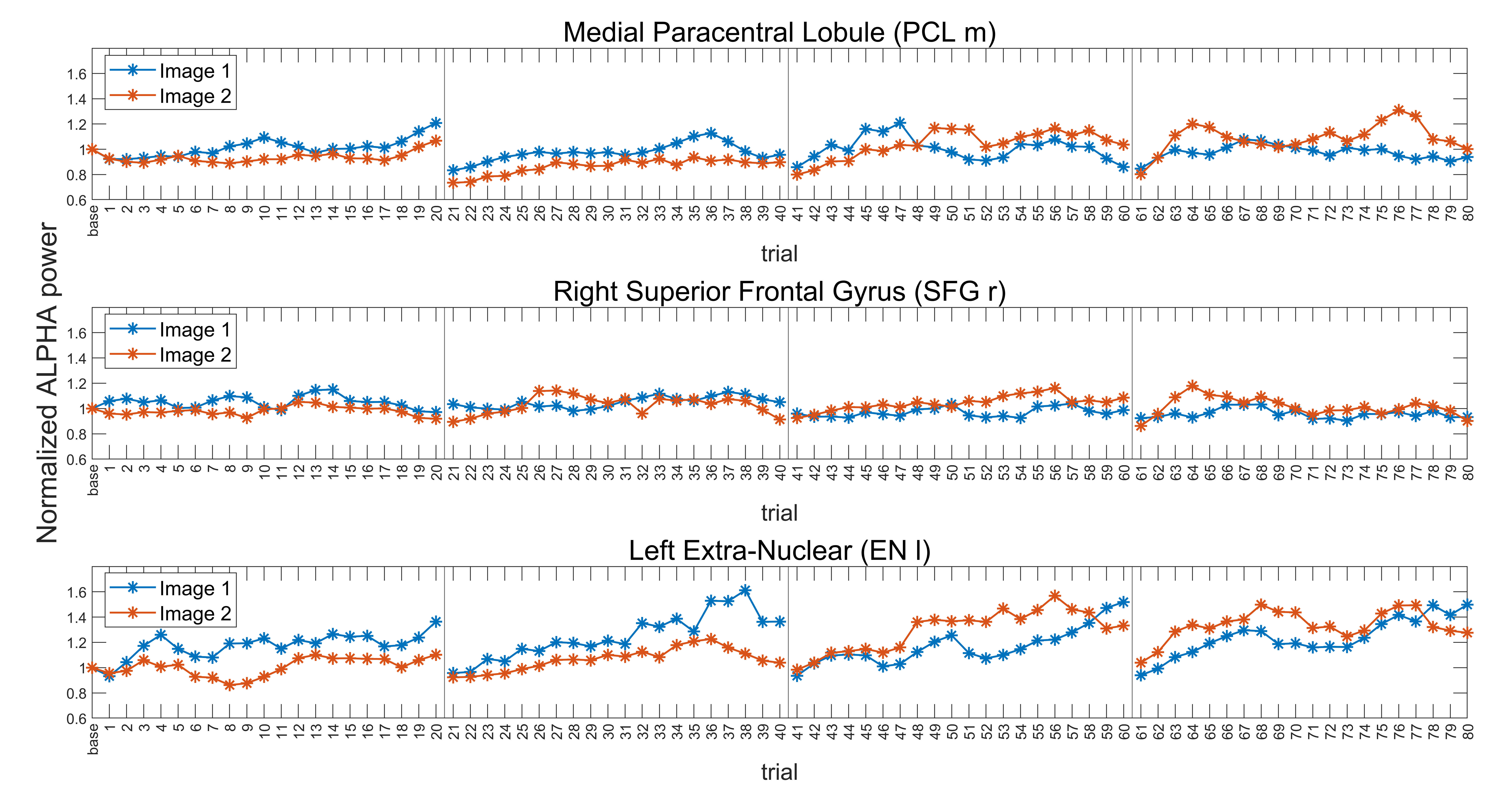


***(SI_6)* Fig. 2** Moving averages (w=3 trials) of normalized alpha power, trial by trial, for the four blocks. The top row shows the normalized power of the medial paracentral lobule (PCL m), the central row the normalized power of the right superior frontal gyrus (SFG r), and the bottom row the normalized power of the left extra-nuclear (EN l). The two images are shown in the same color (blue for Image 1, red for Image 2). Vertical lines are used to delineate the four different blocks.


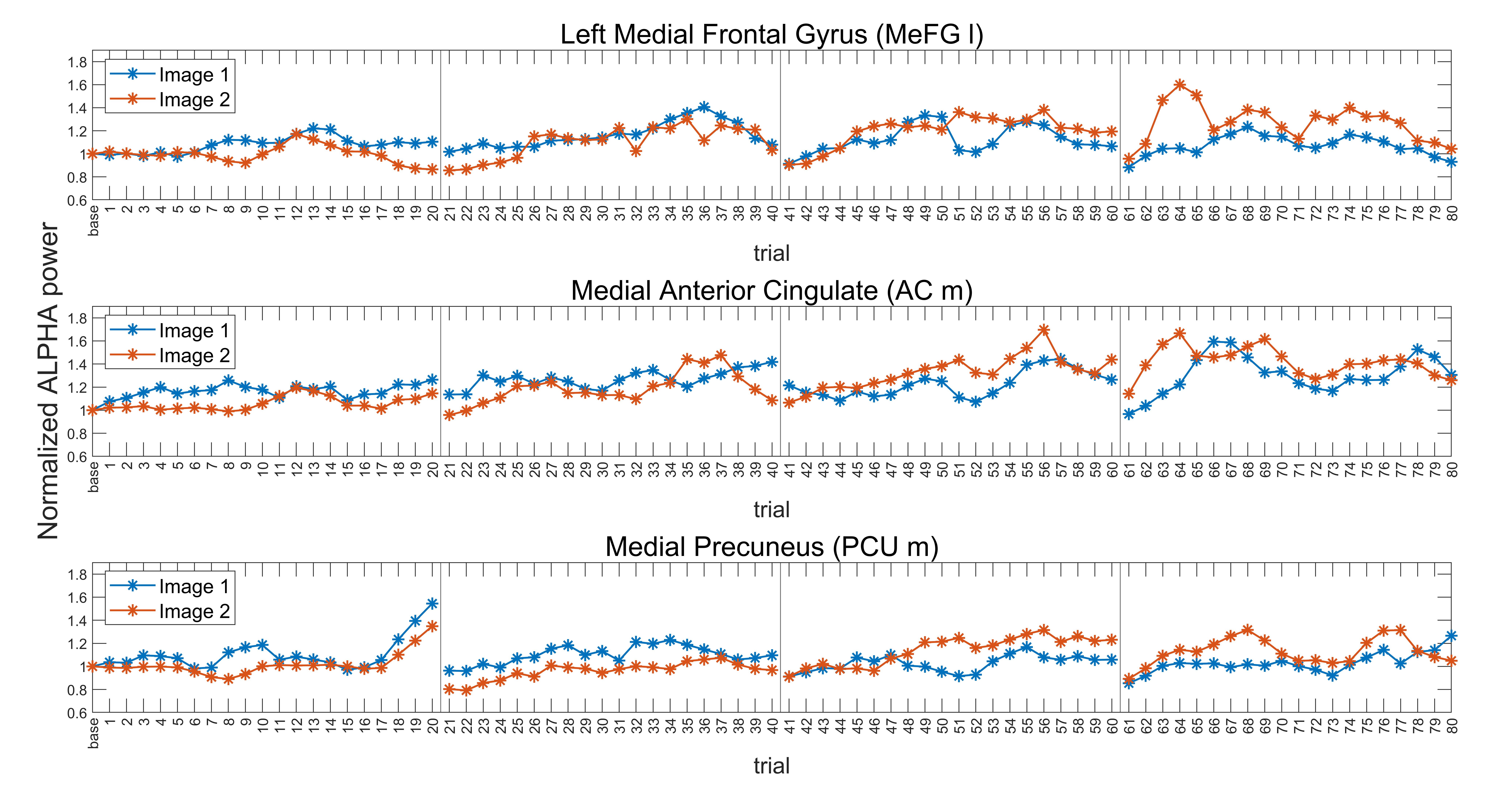


***(SI_6)* Fig. 3** Moving averages (w=3 trials) of normalized alpha power, trial by trial, for the four blocks. The top row shows the normalized power of the left medial frontal gyrus (MeFG l), the central row the normalized power of the medial anterior cingulate (AC m), and the bottom row the normalized power of the medial precuneus (PCU m). The two images are shown in the same color (blue for Image 1, red for Image 2). Vertical lines are used to delineate the four different blocks.
